# Cancer-associated DDX3X mutations drive stress granule assembly and impair global translation

**DOI:** 10.1038/srep25996

**Published:** 2016-05-16

**Authors:** Yasmine A. Valentin-Vega, Yong-Dong Wang, Matthew Parker, Deanna M. Patmore, Anderson Kanagaraj, Jennifer Moore, Michael Rusch, David Finkelstein, David W. Ellison, Richard J. Gilbertson, Jinghui Zhang, Hong Joo Kim, J. Paul Taylor

**Affiliations:** 1 Department of Cell and Molecular Biology, St. Jude Children’s Research Hospital, Memphis, TN 38105, USA; 2 Department of Computational Biology, St. Jude Children’s Research Hospital, Memphis, TN 38105, USA; 3 Department of Oncology, Cambridge Cancer Centre, Cancer Research UK Cambridge Institute, Cambridge, UK; 4 Department of Pathology, St. Jude Children’s Research Hospital, Memphis, TN 38105, USA; 5 Howard Hughes Medical Institute, Chevy Chase, MD 20815, USA

## Abstract

DDX3X is a DEAD-box RNA helicase that has been implicated in multiple aspects of RNA metabolism including translation initiation and the assembly of stress granules (SGs). Recent genomic studies have reported recurrent DDX3X mutations in numerous tumors including medulloblastoma (MB), but the physiological impact of these mutations is poorly understood. Here we show that a consistent feature of MB-associated mutations is SG hyper-assembly and concomitant translation impairment. We used CLIP-seq to obtain a comprehensive assessment of DDX3X binding targets and ribosome profiling for high-resolution assessment of global translation. Surprisingly, mutant DDX3X expression caused broad inhibition of translation that impacted DDX3X targeted and non-targeted mRNAs alike. Assessment of translation efficiency with single-cell resolution revealed that SG hyper-assembly correlated precisely with impaired global translation. SG hyper-assembly and translation impairment driven by mutant DDX3X were rescued by a genetic approach that limited SG assembly and by deletion of the N-terminal low complexity domain within DDX3X. Thus, in addition to a primary defect at the level of translation initiation caused by DDX3X mutation, SG assembly itself contributes to global translation inhibition. This work provides mechanistic insights into the consequences of cancer-related DDX3X mutations, suggesting that globally reduced translation may provide a context-dependent survival advantage that must be considered as a possible contributor to tumorigenesis.

DEAD-box RNA helicases are members of the SF2 superfamily of helicases, which is the largest helicase family containing 37 members in humans, 26 in yeast, and 9 in bacteria (http://www.rnahelicase.org/eukdead.htm). DEAD-box helicases use ATP to remodel RNA-RNA, RNA-DNA or RNA-protein complexes and are known to participate in all facets of the RNA metabolism^1^. Among these proteins, the DDX3X helicase has recently been the focus of intensive study as several recent genomic studies have identified recurrent mutations or chromosome translocations within the DDX3X gene in numerous human tumor types, including T-cell acute lymphoblastic leukemia^2^,^3^, chronic lymphoblastic leukemia^4^,^5^,^6^, natural killer/T-cell lymphomas^7^, head and neck squamous cell carcinomas^8^, lung carcinomas^9^, carcinomas of the breast corpus and uterine endometrium^10^, and medulloblastomas (MB)^11^,^12^,^13^. In MB, DDX3X is mutated in 50% and 11% of pediatric WNT- and SHH-subtypes, respectively^14^; and in 54% of adult SHH-subtype^15^ (matching the frequency of *PTCH1*, the most commonly mutated gene in adult MB). The roles of RNA helicases in the etiology of cancer are largely unknown, and contradictory roles have been ascribed to DDX3X as both an oncogene and tumor suppressor^16^,^17^,^18^. However, various recent studies have reported a positive correlation between high DDX3X levels and poor prognosis in human tumors^19^,^20^, suggesting that overexpression of DDX3X may have oncogenic implications in the etiology of this disease.

According to previous studies, DDX3X and its yeast and *Drosophila* orthologs (Ded1p and Belle, respectively) play multifunctional roles in cells, being implicated in transcription^18^,^21^, splicing^22^,^23^, nuclear export^24^,^25^, and mRNA translation^26^,^27^,^28^,^29^,^30^,^31^. Both DDX3X and its orthologs in other species are prominent components of cytosolic RNA granules, including stress granules (SGs)^25^,^32^,^33^,^34^,^35^. SGs are membrane-less cytosolic bodies composed of mRNAs and proteins that assemble when translation initiation is limiting, and are thought to represent a pool of mRNPs stalled in the process of translation initiation^36^,^37^. Importantly, DDX3X is not only present in SGs but contributes to SG assembly and regulates molecular dynamics within SGs^38^. A growing number of recent studies have revealed that low complexity sequence domains (LCDs) in RNA-binding proteins, including DEAD-box helicases, mediate RNA-protein assemblies such as SGs through the biophysical process of liquid-liquid phase separation^35^,^39^,^40^,^41^,^42^. The central helicase core of DDX3X is flanked by large N-terminal and smaller C-terminal LCDs (Fig. 1a,b). It was recently shown that the N-terminal LCD of LAF-1, the *C. elegans* ortholog of DDX3X, mediates the assembly of germ granules, a structure similar to mammalian SGs^35^, which provides insight into how DDX3X may contribute to SG assembly in mammalian cells.

All reported DDX3X mutations in MB are nonsynonymous single nucleotide variants that lie in its central helicase core, suggesting that these mutations may have deleterious consequences to the normal function of the enzyme (Fig. 1a). In agreement with this line of thought, it was recently reported that two MB-associated DDX3X mutations showed a decreased RNA unwinding activity *in vitro*^43^. In a second study, analysis of two additional MB-associated DDX3X mutants, G302V and G325E, revealed that these mutants are defective in RNA-stimulated ATP hydrolysis, and that introducing these mutations in yeasts result in defective mRNA translation of one previously known mRNA target^44^. However, the breadth of this putative translation defect, and its relevance to human cells expressing MB-associated mutant DDX3X are unknown.

Here, we have used complementary approaches to elucidate how MB-associated mutations interfere with the normal function of DDX3X. We demonstrate that these mutations drive the hyper-assembly of SGs in human cells, leading to impairment in mRNA translation. Additionally, using CLIP-seq, we developed a comprehensive profile of the RNA targets of endogenous DDX3X in human cells. We discovered that DDX3X is enriched in 5′-UTRs proximal to the AUG start site of a specific pool of target mRNAs, and supports their translation. MB-associated mutations do not significantly impact the ability of DDX3X to bind bona fide targets, but translation of these targets is impaired. Surprisingly, based on ribosome profiling, mutant DDX3X expression causes a broad inhibition of translation that impacts DDX3X targeted and non-targeted mRNAs alike. Importantly, this defect can be partially reversed by eliminating the N-terminal low complexity domain of DDX3X or by limiting SG assembly with a genetic approach, suggesting that SG formation mediated by MB-associated mutations contributes to the global translation defect. Thus, our study reveals novel insights into the normal function of DDX3X, uncovers the consequences of MB-associated mutations, and points to a potential strategy for therapeutic intervention for the DDX3X-mediated MB.

## Results

### High levels and punctated distribution of DDX3X in pediatric medulloblastomas and other tumors

**Figure 1:**
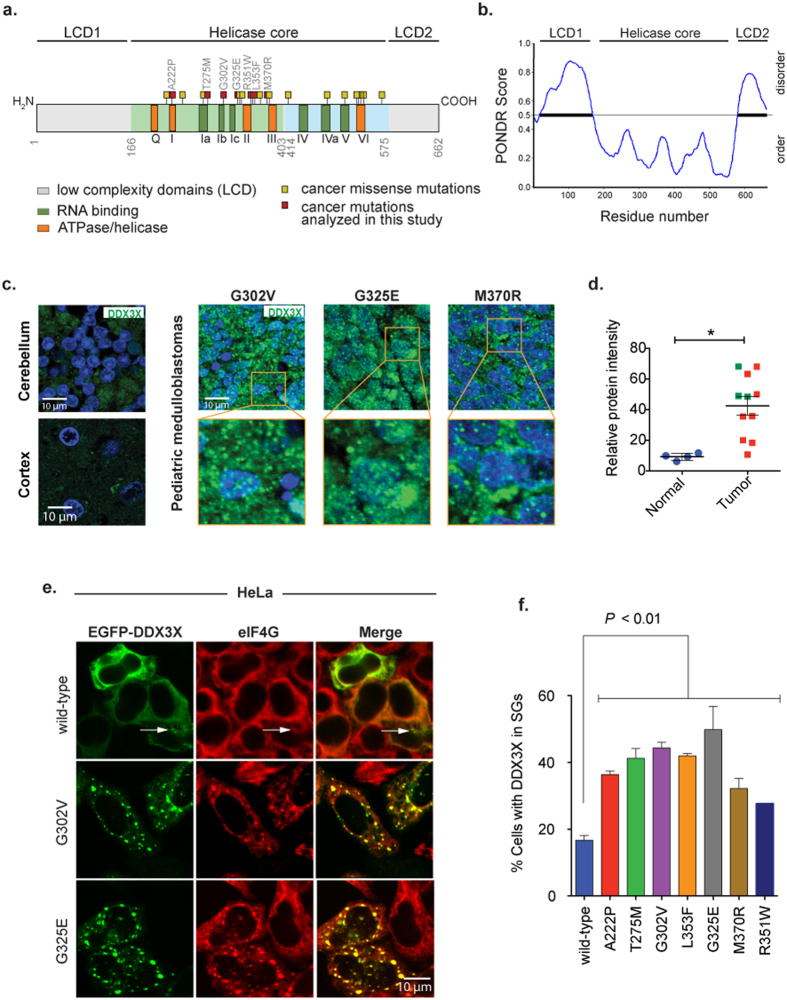
MB-associated mutations in DDX3X drive the assembly of stress granules. (**a**) Schematic representation of DDX3X missense mutation occurring in pediatric MB. Shown is the structural organization of DDX3X protein (ATPase/helicase and RNA binding functional motifs are denoted in orange and dark green boxes; N- and C-terminal low complexity domains are denoted in light gray). MB-associated mutation positions (yellow and red boxes) are based on three genomic studies^11^,^12^,^13^. Cancer mutations analyzed in this study are denoted in the diagram (red boxes). (**b**) Low complexity domains (LCD1 and LCD2) of DDX3X are predicted to be disordered. Scores above 0.5 indicate predicted disorder by the meta-predictor PONDR-FIT^62^. (**c**) Photomicrographs of DDX3X immunofluorescence in three pediatric MBs carrying mutations in DDX3X and normal brain (cerebellum and cortex). Green and blue colors represent DDX3X and DAPI staining, respectively. Boxes denote magnified area shown at the bottom of the indicated sample. (**d**) Quantification of protein levels in MB tumors and normal brain. Green squares represent tumors carrying mutations in DDX3X (panel c), while red represent tumors carrying the wild-type form. Mean ± SEM is shown. **P = 0.0068, Student’s *t*-test comparison between normal brain tissues and MB tumors. (**e**) Co-localization of the indicated EGFP-tagged DDX3X with the SG marker eIF4G in HeLa cells under normal culture conditions. EGFP-tagged DDX3X plasmids were transfected in HeLa cells for 24 h followed by immunofluorescence against the SG marker eIF4G (red). (**f**) Quantification of SGs in HeLa cells transfected with wild-type and MB-associated mutant DDX3X forms for 24 h under normal conditions. Mean ± SEM values are based on a minimum of three replicated experiments. Student’s *t*-test comparison between wild-type DDX3X and each MB-associated mutants is shown.

To investigate the expression level of DDX3X in pediatric MB, we performed immunofluorescence against DDX3X in a panel of these tumors with or without DDX3X mutations. Whereas DDX3X expression was relatively low in normal cerebellar or cortical tissue, DDX3X expression showed frequent up-regulation regardless of its mutational status in MB tumors (Fig. 1c,d, and Supplementary Figure S1a), consistent with prior reports in other tumor types^19^,^20^. All 3 MB samples carrying DDX3X mutations showed a significant increase in DDX3X expression relative to normal brain. Interestingly, we observed a cytoplasmic punctated distribution of DDX3X in these tumors (Fig. 1c and Supplementary Figure S1a). We also used a publically available database (http://www.proteinatlas.org/)^45^ to investigate the expression levels and cellular distribution of DDX3X in other tumor types and observed a similar high levels and punctate distribution in a wide range of human cancers including glioma, melanoma and breast carcinoma tumors compared to normal tissues (Supplementary Figure S1b). Thus, these data suggest that high DDX3X expression in a punctate distribution is frequent in human tumors.

### MB-associated DDX3X mutations drive the hyper-assembly of stress granules

The punctated distribution of DDX3X in human tumors prompted us to investigate the subcellular localization of MB-associated DDX3X mutants in cell lines. Epitope-tagged wild-type DDX3X was expressed diffusely in the cytoplasm with rare accumulation in cytoplasmic puncta in HeLa cells (Fig. 1e). This subcellular expression pattern was consistent with the distribution of endogenous DDX3X in HeLa cells as well as in lower rhombic lip progenitor (LRLP) cells, which are the cells of origin of WNT-subtype MB (Supplementary Figure S2a). In contrast, despite comparable or lower levels of expression, MB-associated DDX3X mutants were found to assemble in discrete cytoplasmic puncta with significantly greater frequency than wild-type DDX3X (Supplementary Figure S2b, Fig. 1e). Immunofluorescence staining for the SG marker eIF4G confirmed that DDX3X mutants accumulated in SGs (Fig. 1e). Quantitative analyses showed that MB-associated mutations in DDX3X drove SG assembly to a significantly greater extent than wild-type in the absence of stress (Fig. 1f). To eliminate the potential contribution of transient transfection, which may result in DDX3X expression to a greater extent than endogenous levels, we generated *Tet-On*-inducible HEK293T cells expressing wild-type or the MB-associated mutant DDX3X G325E (Supplementary Figure S2c). When exogenous DDX3X expression was induced comparable to endogenous levels, wild-type DDX3X remained diffuse in the cytoplasm, whereas mutant DDX3X accumulated in SGs (Supplementary Figures 2d,e) under normal culture conditions, confirming significant mutation-dependent augmentation of SG assembly. Both, wild-type or the MB-associated mutant DDX3X were almost completely relocalized to SGs under stress conditions (Supplementary Figure 2d,e). Finally, to investigate the impact of mutations on DDX3X stability, we examined protein half-life using our *Tet-on*-inducible HEK293T cells. We observed that MB-associated mutant DDX3X G325E shows comparable or modestly reduced stability relative to wild-type DDX3X, consistent with comparable or modestly reduced steady state levels of mutant DDX3X (Supplementary Figure 2f). Thus, SG assembly mediated by MB-associated mutations in DDX3X is not a consequence of a greater stability of the protein.

### MB-associated DDX3X mutations bind RNA and impair mRNA translation

The presence of MB-associated mutant DDX3X in SG implies that these mutants retain the ability to bind RNA. However, it has been proposed that these mutations would interfere with normal nucleic acid binding capabilities based on structural modeling predictions^12^. Thus, we next sought to examine the impact of MB-associated DDX3X mutations on RNA binding in intact cells. We performed immunoprecipitation (IP) of wild-type or mutant DDX3X that had been ultraviolet (UV) cross-linked to its native RNA targets. We included two additional mutants in our analysis: a point mutation within the DEAD motif of DDX3X (DDX3X^DQAD^), which is expected to impair the helicase/ATPase activities of the protein and to prevent release of ATP hydrolysis products^46^,^47^, and a deletion mutant within motif IVa (DDX3X^∆IVa^), which is expected to impair nucleic acid binding capabilities^48^. As expected, DDX3X^DQAD^ showed enhanced RNA-binding capacity relative to wild-type DDX3X, whereas DDX3X^∆IVa^ displayed impaired ability to bind RNA (Supplementary Figure S3a–c). Consistent with a role in driving SG hyper-assembly, which implies retained RNA-binding ability, we found that MB-associated DDX3X mutants effectively precipitated target RNAs in amounts similar or only modestly reduced relative to wild-type DDX3X (Supplementary Figure S3a–c).

**Figure 2:**
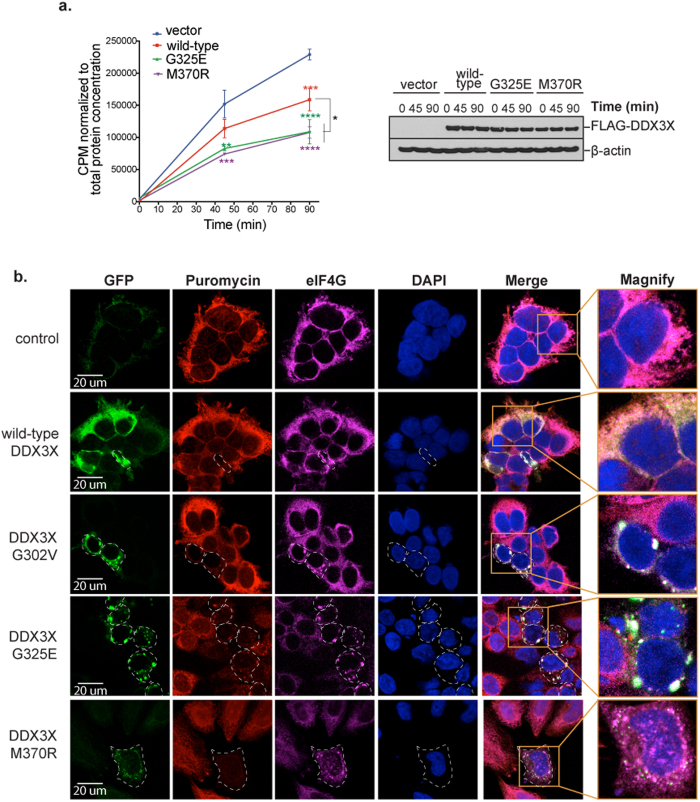
MB-associated DDX3X mutations impair mRNA translation. (**a**) ^35^S-Met/Cys labeling of nascent polypeptides in HEK293T cells transfected for 36 h with the indicated FLAG-tagged DDX3X variants or empty vector (control). Values are the mean counts per minute (CPM) of a minimum of three replicates per sample, normalized to total protein level as a function of labeling time (min) with ^35^S-Met/Cys. Error bars represent SEM at each time point. Two-way ANOVA: *P ≤ 0.05; **P ≤ 0.01; ***P ≤ 0.001; ****P ≤ 0.0001. Western blot (right) shows DDX3X levels in pooled replicates at each time point; β-actin served as the loading control. (**b**) Puromycin incorporation analysis in HEK293T cells expressing EGFP-tagged wild-type or cancer-related mutant DDX3X (G302V, G325E, M370R). Puromycin incorporation was conducted as indicated in Material and Methods. Translation is monitored by staining against puromycin (red), while SGs are detected by staining against eIF4G (magenta). Green color represents EGFP-tagged DDX3X, nuclei are stained with DAPI. Dashed lines denote cells with impaired translation as monitored by lack of puromycin; yellow boxes show magnified area at the right.

Since SG assembly typically correlates with translational impairment^49^, we next sought to determine whether the SG induction by MB-associated DDX3X mutations was associated with altered translation. First, we assessed the impact of DDX3X on translation by ^35^S-Met/Cys metabolic labeling. Over-expression of wild-type DDX3X resulted in modest impairment of translation consistent, and this observation was concordant with the low rate of SG assembly initiated by over-expression of wild-type DDX3X (Figs 1e,f and 2a). By contrast, over-expression of DDX3X mutants, G325E and M370R, at equivalent levels resulted in significantly greater translation impairment (Fig. 2a), correlating with the high frequency of SG assembly driven by these proteins (Fig. 1e,f). Second, we monitored mRNA translation in cells expressing wild-type or mutant DDX3X by pulse-labeling with puromycin for 30 minutes. Puromycin immunostaining permits visualization of newly synthesized proteins^50^ and the relationship to SG assembly in individual cells. This analysis showed a striking correlation between assembly of SGs and profound impairment of translation, which was significantly more frequent when DDX3X mutants where expressed (Fig. 2b). Thus, we consistently observe impairment in mRNA translation associated with MB-associated mutations in DDX3X by two independent assays.

### DDX3X binds predominantly to mature mRNAs

The translation defect mediated by MB-associated mutations in DDX3X was anticipated based on the finding that (i) mutant DDX3X binds RNA and induces SG hyper-assembly (Fig. 1 and Supplementary Figure S3), and (ii) these mutants are defective in RNA-stimulated ATPase-activity^44^. What was surprising, however, was the apparently broad inhibition of translation as revealed by two distinct metabolic labeling approaches given the presumed limited role of DDX3X in facilitating translation of a discrete subset of mRNA targets harboring long and highly structured 5′-UTRs^25^. This paradox motivated a comprehensive investigation of DDX3X RNA targets by CLIP-seq^51^.

**Figure 3:**
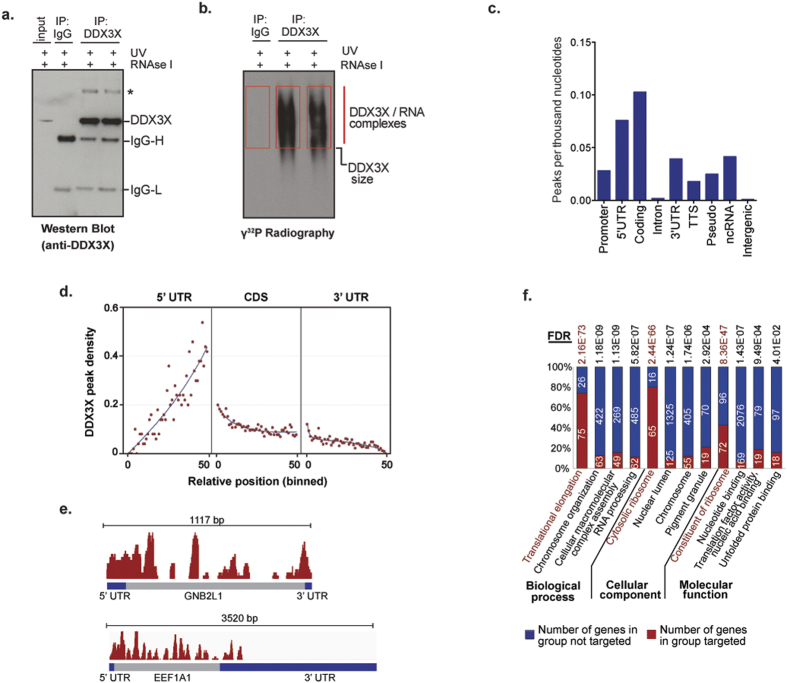
CLIP-seq of endogenous DDX3X identified RNA targets. (**a**) Western blot analyses of immunoprecipitated endogenous DDX3X used in the CLIP-seq experiment. A higher-mobility protein band (indicated by *) of 150 kDa represents an oligomeric form of DDX3X identified by mass spectrometry (data not shown). (**b**) Radiography of γ^32^P-labeled DDX3X/RNA complexes migrating near the size of DDX3X. RNA material extracted for CLIP-seq is denoted by red boxes. (**c**) Peak density, represented by number of peaks per Kb to annotated genomic regions (TTS, transcription termination sites; Pseudo, pseudogenes; ncRNA, non-coding RNA; 5′-UTR, 5′ untranslated regions; 3′-UTR, 3′ untranslated regions; CDS, coding sequences). (**d**) Metagene analysis of DDX3X binding density across coding and non-coding regions of mRNA targets. To normalize differences in gene size, 5′-UTR, CDS and 3′-UTR (marked at the top) for each gene is divided into 50 bins, with 0 and 50 corresponding to the start and end of each region, respectively. The number of peaks in each bin was summarized across all genes and the density marks the number of peaks per 1Kb in each bin. (**e**) Examples of DDX3X CLIP targets. Coding sequences for GNB2L1 and EEFA1 mRNAs are shown in gray (intronless). Blue color denotes 5′- and 3′-UTRs. Red color above the mRNA structure denotes DDX3X binding to mRNAs. (**f**) Gene ontology analyses of DDX3X CLIP-seq mRNA targets obtained by DAVID Bioinformatics Resource 6.7. False discovery rate (FDR) is shown for each category.

First, we examined the specificity and efficiency of 5 different anti-DDX3X antibodies for IP followed by mass spectrometry (MS) of the recovered protein to confirm the specificity of the interaction (Supplementary Figure S4a,b). The best of these antibodies was used for IP of endogenous DDX3X that had been cross-linked to its native RNA targets by UV irradiation. Recovery of endogenous DDX3X was monitored by Western blot (Fig. 3a), whereas recovery of cross-linked target RNA was monitored by autoradiography after RNase treatment and end-labeling with γ^32^P (Fig. 3b). Recovered RNA was amplified and sequenced as previously described^51^. DDX3X CLIP-seq was performed in duplicates and a control experiment using IgG antibody that does not recognize DDX3X was included to monitor background signal. From each DDX3X CLIP experiment, we obtained 2.4–2.6 × 10^6^ mapped reads in the DDX3X CLIP-seq data set compared to <18,000 in control, showing high signal to noise ratio in the CLIP-seq data (Supplementary Figure S4c). Peak calling was performed to identify DDX3X target RNAs (the complete list of DDX3X target RNAs is included in Supplementary Table S1). Two biological replicates showed high reproducibility (Pearson correlation coefficient 0.934, Supplementary Figure S4d), therefore these data were pooled for subsequent analyses.

Assessment of DDX3X peak density across defined genomic regions revealed that DDX3X binding was enriched in coding regions as well as the 5′- and 3′-UTRs, and exhibited very low binding to intronic and intergenic regions (Fig. 3c). The very high enrichment in exonic compared to intronic regions suggests that DDX3X primarily binds to mature mRNAs. Metagene analysis of DDX3X occupancy showed that within mRNA species, DDX3X binding spanned the entire transcript with a prominent enrichment in the 5′-UTR proximal to the AUG start codon (Fig. 3d,e, Supplementary Figure S4e). This binding pattern of DDX3X at 5′-UTR suggests a role for DDX3X in assisting the ribosome during late events of translation initiation, whereas binding to coding regions suggests that this helicase may also participate in subsequent elongation steps.

Standard motif-finding algorithms failed to identify a favored binding motif within the mRNA targets, suggesting that either DDX3X recognizes RNA structural features rather than a primary nucleotide sequences or that the association of DDX3X with protein partners may also account for the positional preferences of this helicase towards its target mRNAs. While DDX3X binds to abroad range of mRNA targets, gene ontology analyses using DAVID illustrated that DDX3X targets were significantly enriched in genes encoding proteins involved in RNA metabolism, particularly the process of translation, although other cellular processes such as viral reproduction, chromosome organization, and cellular trafficking were also represented (Fig. 3f and Supplementary Table S2).

### DDX3X regulates the translation of its mRNA targets

**Figure 4:**
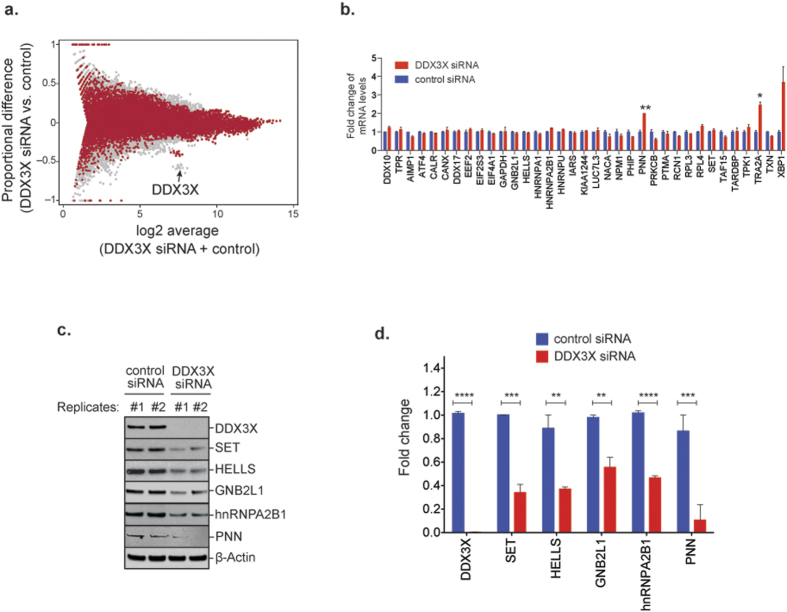
DDX3X regulates its mRNA targets by modulating their translation, not their levels. (**a**) MA (log ratio vs. abundance) plot comparing expression profiles of HEK293T cells treated with control or DDX3X siRNAs. FPKM values from RNAseq data was used in this analysis. DDX3X targets are represented as red dots, while non-target RNAs are represented as gray dots. Efficient knockdown is confirmed by detection of low mRNA levels of DDX3X (denoted by black arrow). (**b**) Reverse transcription-qPCR analysis of 33 DDX3X CLIP-seq targets plus two non-targets (DDX10 and TPR) in HEK293T cells treated with control or DDX3X siRNAs. GAPDH was used to normalize the samples. Mean ± SEM values are based on a minimum of two replicated experiments. Only two putative targets showed significantly altered mRNA levels (student’s *t*-test comparison: *P = 0.01; **P = 0.008). (**c**) Western blot analysis of proteins encoded by DDX3X-targeted mRNAs (SET, HELLS, GNB2L1, hnRNPA2B1, PNN) identified by CLIP-seq in HEK293T cells treated with control or DDX3X siRNAs. β-actin was used as the loading control. Reduced protein expression is observed for DDX3X and its targets. (**d**) Quantification of relative protein levels in HEK293T cells treated with control or DDX3X siRNAs. Values are the means of two biological replicates. Error bars represent SEM. Student *t*-test comparisons: **P ≤ 0.01; ***P ≤ 0.001; ****P ≤ 0.0001.

With the identity of DDX3X target mRNAs in hand for the first time, we next sought to elucidate the role of DDX3X in the metabolism of these mRNAs. Our assessment began with RNAseq-based expression profiling in HEK293T cells with or without RNAi-mediated knockdown of endogenous DDX3X. Depletion of endogenous DDX3X had no significant impact on the expression level or splicing of DDX3X mRNA targets (red) and non-targets (grey) (Fig. 4a), a result we confirmed by quantitative PCR analysis for 33 individual RNA targets of DDX3X (Fig. 4b). In contrast, we found that the steady state levels of the proteins produced by DDX3X-targeted mRNAs was consistently and significantly reduced in DDX3X depleted cells (Fig. 4c,d). This observation strongly implies that DDX3X positively regulates expression of its target mRNAs at the levels of translation.

### MB-associated mutations in DDX3X impair the translation of its mRNA targets

**Figure 5:**
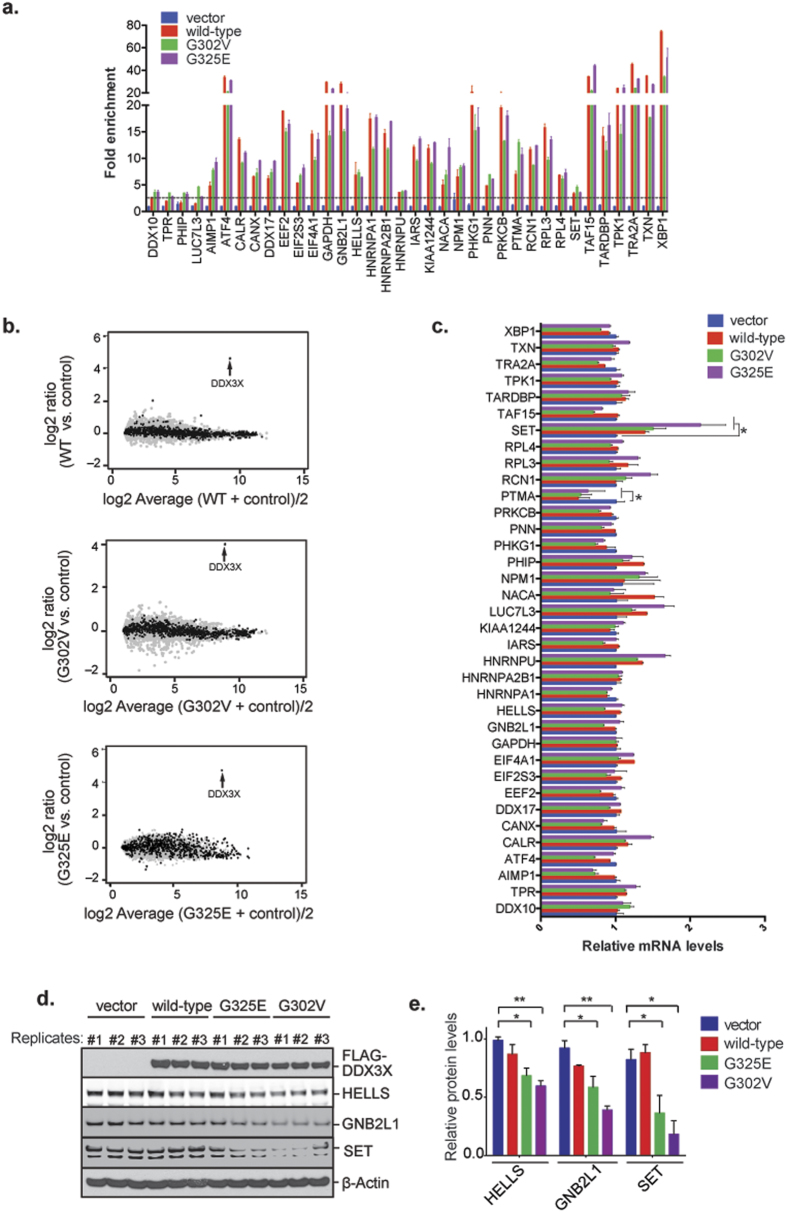
MB-associated mutations in DDX3X impact the translation of mRNA targets. (**a**) RNA-immunoprecipitation analysis of numerous DDX3X targets using exogenous FLAG-tagged wild-type DDX3X and two cancer mutants (G302V and G325E). Two non-target RNAs were analyzed as negative control (DDX10 and TPR). Cells transfected with empty vector and immunoprecipitated with the anti-FLAG antibody M2 served as control. 87.5% of DDX3X mRNA targets were validated by the assay (horizontal black-dotted line represents cut-off based on non-target RNAs). (**b**) MA plot comparing RNA-seq data sets from HEK293T cells transfected with empty vector or a DDX3X-expressing vector (wild-type and two mutants: G302V and G325E). Transfections efficiencies are confirmed by the detection of high mRNA levels of DDX3X itself (denoted by the black arrows). Black dots represent DDX3X targets, gray dots represent non-targets. (**c**) Reverse transcription–qPCR analysis of several DDX3X CLIP-seq targets plus two non-targets (DDX10 and TPR) in HEK293T cells in cells expressing either wild-type DDX3X or two cancer-related mutant (G302V and G325E). Mean ± SEM values are based on a minimum of two replicated experiments. ~91% of DDX3X mRNA targets showed insignificant changes in their levels between cells expressing vector and those expressing DDX3X variants (Multifactorial ANOVA: *P ≤ 0.01). (**d**) Western blot analyses against FLAG and CLIP-seq targets HELLS, GNB2L1, and SET in HEK293T cells transfected with FLAG-tagged wild-type DDX3X or two cancer-related mutants (G325E and M370R). (**e**) The mean protein levels of three biological replicates of samples shown in (**d**) was graphed. Error bars represent mean values ± SEM (Student’s *t*-test between control or cells expressing DDX3X variants; *P ≤ 0.05; **P ≤ 0.01).

We next assessed the ability of DDX3X mutants to bind 34 specific target RNAs using IP followed by qPCR (RIP-qPCR). Consistent with the data indicating that mutant DDX3X retains RNA-binding ability (Supplemental Figure S3), this analysis confirmed that mutant DDX3X bound well to specific target mRNAs. With rare exception, target binding by DDX3X mutants did not differ significantly from target binding by wild-type DDX3X (Fig. 5a). We also evaluated the consequence of mutant DDX3X on the stability of its target RNAs by performing RNA-seq and comparison of exon-level expression in HEK293T cells transfected with wild-type or mutant DDX3X. Similar to our observations with depletion of endogenous DDX3X, over-expression of neither wild-type nor mutant DDX3X had a significant impact on the expression levels of mRNA targets of DDX3X (black) or non-targets (grey) (Fig. 5b). This result was confirmed by quantitative PCR analysis of 34 individual RNA targets of DDX3X revealed only rare changes in expression level in association with mutant DDX3X, with modest down-regulation of one mRNA (PTMA) (Fig. 5c). Thus, mutant DDX3X retains the ability to interact with target mRNAs and this does not significantly alter the expression levels of these targets. In stark contrast, over-expression of mutant but not wild-type DDX3X resulted in consistently and significantly reduced steady state levels of proteins produced by DDX3X target mRNAs (Fig. 5d,e). These results indicate that DDX3X mutations do not significantly impair RNA binding or stability, but impair translation of its target mRNAs.

### Ribosome profiling illustrates that MB-associated mutations in DDX3X impair translation initiation of both targeted- and non-targeted mRNAs

**Figure 6:**
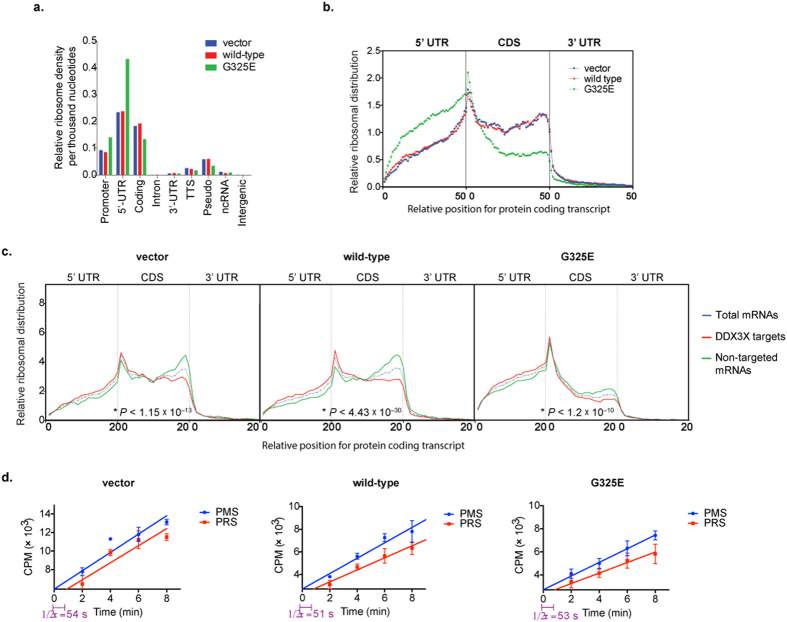
Ribosome profiling illustrates that expression of MB-associated DDX3X mutant G325E results in accumulation of ribosomes at 5′-UTR of mRNAs and impairs global translation. (**a**) Ribosomal density per thousand nucleotides uniquely mapped to various annotated genomic regions (TTS, transcription termination sites; Pseudo, pseudogenes; ncRNA, non-coding RNAs; 5′-UTR, 5′- untranslated regions; 3′-UTR, 3′ untranslated regions; CDS, coding sequences). (**b**) Metagene analyses of ribosomal densities across the mRNA structure. Shown are the relative ribosomal density curves calculated for each of the 50-binned positions among three regions of the mRNA (5′-UTR, CDS, and 3′-UTR). Vertical lines separate the three regions of the mRNA. (**c**) MB-associated DDX3X mutant G325E impairs global translation. Metagene analyses of ribosomal densities across the mRNA structure for total mRNA (dashed-blue line), mRNAs identified as DDX3X targets (red lines), and mRNAs not identified as DDX3X targets (green lines) by CLIP-seq. The analysis was done in control cells or cells expressing wild-type DDX3X or the G325E mutant form. Shown are the relative ribosomal density curves calculated for each of the 20-binned positions among three regions of the mRNA (5′-UTR, CDS, and 3′-UTR). Vertical lines separate the three regions of the mRNA. The *P*-values for significant changes of ribosomal distribution between target or non-target mRNAs in the coding sequence was calculated using Kolmogorov-Smirnov equality-of-distribution test. (**d**) Ribosome half-transit analyses for cells expressing vector or FLAG-tagged wild-type DDX3X or MB-associated mutant DDX3X-G325E for 24 h in HEK293T cells. ^35^S-Met/Cys labeling incorporation into all polypeptides (postmitochondrial supernatant, PMS) and into polypeptide released from ribosomes (postribosomal supernatant, PRS) was obtained by linear regression analysis. Mean ± SEM values are based on a minimum of four replicated experiments. Ribosome half-transit time (1/2τ) were relatively equal in all samples.

To deeply investigate the translation defect caused by DDX3X mutations we employed ribosome profiling in cells expressing wild-type or mutant DDX3X. Combined with knowledge of DDX3X targeted mRNAs as revealed by CLIPseq, ribosome profiling permits assessment of DDX3X mutation on the translation of individual targeted and non-targeted mRNAs. Ribosome profiling involves purification and quantitative assessment of RNA fragments bound and protected by ribosomes in intact cells, which yields high resolution ‘footprints’ of ribosomes on actively translating messages^52^. Thus, we transfected HEK293T cells with empty vector, wild-type or mutant DDX3X, followed by isolation and high-throughput sequencing of ribosome-protected mRNA fragments (Supplementary Figure S5a). As expected, ribosome binding was highly enriched in 5′-UTRs and coding regions (Fig. 6a). The obtained ribosome profiles had additional characteristics consistent with accurate capture of bone fide ribosome-protected mRNA fragment, such as elevated ribosome density at the beginning and end of coding sequences, reflecting slower ribosome processivity at initiation and termination sites, and rapid drop-off in ribosome densities immediately after termination codons^53^.

We performed metagene analysis to analyze ribosome occupancy on a global scale. This analysis showed minimal difference in ribosome occupancy profiles between cells expressing empty vector and those expressing wild-type DDX3X (Fig. 6b), whereas cells expressing mutant DDX3X showed a significant decrease in ribosomal density within coding regions relative to cells expressing empty vector or wild-type DDX3X, consistent with a substantial global defect in translation (Fig. 6b). Surprisingly, we noted that expression of mutant DDX3X impacted targeted and non-targeted mRNAs alike, consistent with the broad impact of mutant DDX3X on translation (Fig. 6c).

Concomitant with altered ribosome density in the coding regions in cells expressing mutant DDX3X there was also a striking increase in ribosomal density in the 5′-UTR relative to cells expressing empty vector or wild-type DDX3X (Fig. 6b and Supplementary Figure S5b). This pattern of ribosome occupancy suggests mutation-dependent impairment of a DDX3X function in late events translation initiation, consistent with the occupancy pattern of mutant DDX3X on target mRNAs (Fig. 3d). Whereas these results strongly suggest a mutation-dependent defect in translation initiation, a failure of translation elongation could also contribute to the global impairment in protein synthesis we observed. To rule out this possibility, we examined ribosome half-transit times, which reflect translation elongation efficiency. This analysis showed that expression of mutant DDX3X did not significantly affect elongation rates compared to cells expressing wild-type DDX3X or vector control, suggesting no mutation-dependent defect in translation elongation (Fig. 6d).

### Removing the N-terminal low complexity domain of DDX3X prevents cancer-associated mutant from inducing SG assembly and repressing mRNA translation

**Figure 7:**
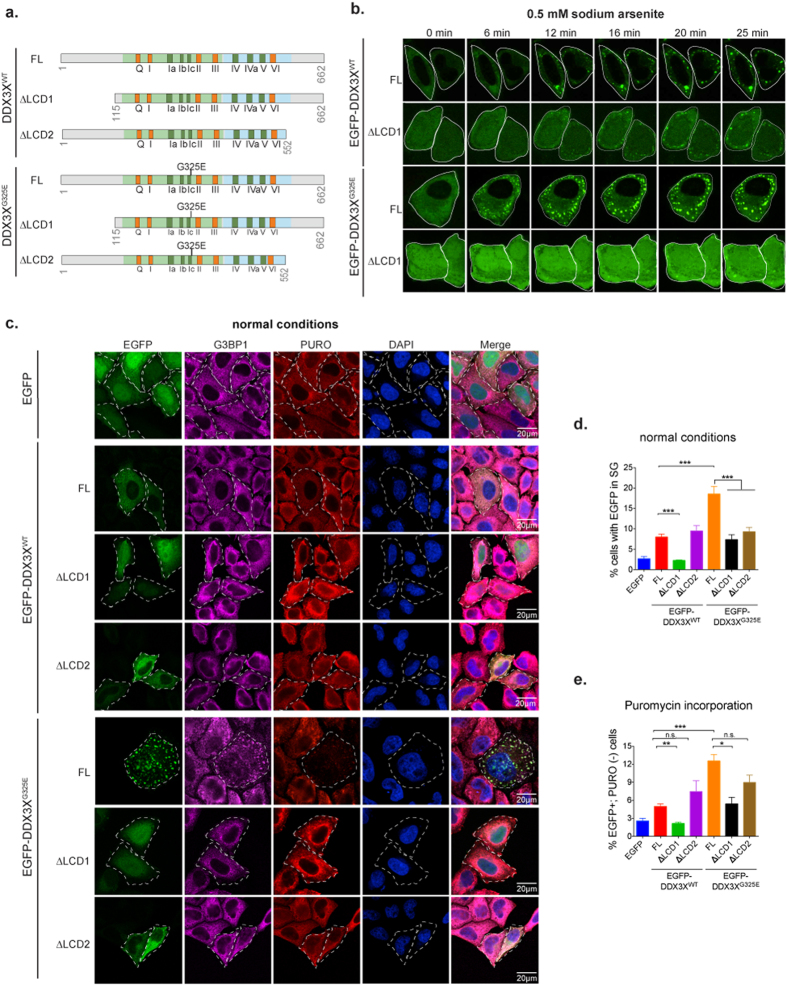
Deletion of N-terminal low complexity domain prevents cancer-related DDX3X mutant from inducing SG assembly and repressing mRNA translation. (**a**) Schematic representation of full-length DDX3X or DDX3X with deletion of either N-terminal low complexity domain lacking amino acids 1–114 (ΔLCD1) or C-terminal low complexity domain lacking amino acids 553–662 (ΔLCD2). The ATPase/helicase and RNA binding functional motifs are denoted in orange and dark green boxes. The location of the MB-associated mutant G325E is shown in the scheme. (**b**) Live imaging of HeLa cells transfected with the indicated EGFP-tagged DDX3X constructs for 24 h and treated with sodium arsenite for a period of 25 min. Images were collected every 30 seconds. Representative images of the indicated time-points are shown. (**c**) Puromycin incorporation assay in HeLa cells transfected with EGFP alone or the indicated EGFP-tagged DDX3X constructs for 24 h. After pulse labeling of newly synthesized proteins using puromycin for 30 min, cells were fixed and immmunostained against G3BP1 (magenta) and puromycin (PURO, red). EGFP signal was used to visualize the desired tagged proteins (denoted by white curve lines). Nuclei were stained with DAPI (blue). (**d**) Quantification of SGs in HeLa cells transfected with EGFP alone or the indicated EGFP-tagged DDX3X constructs for 24 h under normal conditions. Mean ± SEM values are based on a minimum of three replicated experiments. Student’s *t*-test comparison between full-length DDX3X and either ΔLCD1 or ΔLCD2 mutants is shown (***P ≤ 0.001). (**e**) Quantification of cells expressing EGFP plasmids (EGFP+) that are concomitantly devoid of PURO incorporation [PURO(−)] from the experiment shown in (**c**). Mean ± SEM values are based on a minimum of three biological replicates. Student’s *t*-test comparison between full-length DDX3X and either ΔLCD1 or ΔLCD2 mutants is shown (*P ≤ 0.05; **P ≤ 0.01; ***P ≤ 0.001; *n.s.*, not significant).

Ribosome profiling indicates that cancer-associated mutation impacted the translation of DDX3X targeted and non-targeted mRNAs alike (Fig. 6), consistent with the global impairment of translation revealed by metabolic labeling studies (Fig. 2). The perfect correlation between SG formation and global translation impairment at the level of single cell resolution (Fig. 2b) suggested that SG assembly itself could be the basis of translation impairment that extends beyond DDX3X targets, by sequestering non-target mRNAs and/or essential translation factors in SGs. To test this hypothesis we took advantage of recent insights into the contribution of low complexity domains (LCDs) to the assembly of SGs and related non-membrane bound organelles^35^,^39^,^40^,^41^,^42^. The central helicase core of DDX3X is flanked by large N-terminal and smaller C-terminal LCDs (Fig. 1a). Deletion of the N-terminal LCD of LAF-1, the *C. elegans* ortholog of DDX3X, significantly impedes the assembly of germ granules, a structure similar to mammalian SGs^35^. Thus, we engineered EGFP-tagged wild-type and mutant DDX3X lacking either N- or C-terminal LCDs (Fig. 7a). Immunofluorescence of HeLa cells transfected with these DDX3X constructs revealed that whereas the full-length proteins are efficiently recruited to SGs in response to oxidative stress, deletion of N-terminal LCD (ΔLCD1) significantly limited the recruitment of these proteins to SG under these conditions (Supplementary Figure S6a,b). Deletion of C-terminal LCD (ΔLCD2) however, showed limited impact on SG assembly (Supplementary Figure S6a,b), similar to observations with LAF-1^35^. To further analyze the requirement of LCD1 in driving SG assembly of DDX3X, we performed live imaging in HeLa cells treated with sodium arsenite for a period of 25 min. This analysis confirmed that deletion of LCD1 limited the recruitment of both wild-type and G325E-DDX3X to SGs in response to oxidative stress (Fig. 7b). Quantitative analyses confirmed that deleting LCD1 of DDX3X significantly prevented SG hyper-assembly mediated by both wild-type DDX3X and the MB-associated mutant G325E under normal and stress conditions (Fig. 7c,d). Importantly, deletion of LCD1 also reversed the translation defect that accompanied DDX3X-depended SG formation as monitored by puromycin incorporation (Fig. 7c,e). These results suggest that SG hyper-assembly contributes to the global translation defect caused by cancer-associated DDX3X mutations.

### SG hyper-assembly mediated by MB-associated DDX3X mutations are independent of eIF2α phosphorylation

A possible mechanism whereby MB-associated mutations in DDX3X could induce SG assembly and affect global translation is induction of a stress response, perhaps through accumulation of abnormal mRNA, leading to phosphorylation of eukaryotic translation initiation factor alpha (eIF2alpha) and activation of an integrated stress response that includes SG assembly and global translation impairment^54^. To investigate this possibility, we treated HeLa cells with the small molecule inhibitor of the integrated stress response, ISRIB, which potently inhibits stress granule assembly downstream of eIF2alpha phosphorylation^55^. Whereas ISRIB significantly inhibited SG assembly induced by arsenite treatment (Supplementary Figure S7a,b), it did not prevent the formation of SG initiated by MB-associated DDX3X mutants (Supplementary Figure S7a,c). Similarly, the PERK inhibitor GSK2606414^56^ did not prevent the formation of SGs mediated by MB-associated mutations (Supplementary Figure S8). Together, these data indicate that the initiation of SG assembly by DDX3X is independent of eIF2alpha phosphorylation.

### Depletion of the SG assembly factors G3BP1/G3BP2 prevents DDX3X-induced SG formation and allows normal rates of mRNA translation

**Figure 8:**
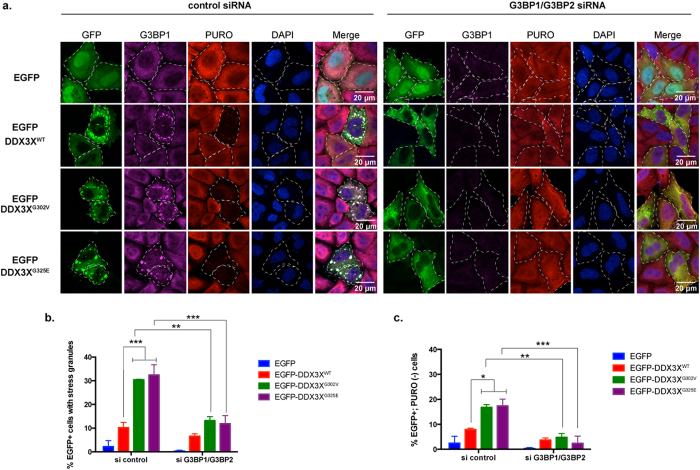
Knocking-down the SG nucleator factors G3BP1/G3BP2 prevents DDX3X-induced SG formation and allows normal rates of mRNA translation. (**a**) Puromycin incorporation assay in HeLa cells treated with control siRNAs (left panel) or G3BP1 and G3BP2 siRNAs (right panel) for 24 h followed by transfection with the indicated EGFP plasmids for another 24 h. Immunofluorescence was performed against G3BP1 (magenta) and puromycin (PURO, red). GFP signal was used to visualize the desired tagged proteins. Shown are representative images for each sample. Dashed lines denote cells displaying DDX3X granules and are devoid of PURO incorporation. Nuclei were stained with DAPI (blue). (**b**) Quantification of SG formation in cells expressing EGFP (control) or EGFP-DDX3X variants (wild-type, G302V, or G325E) from the experiment shown in (**a**). Error bars represent mean values ± SEM (*N* = 3, Two-way ANOVA; **P ≤ 0.01, ***P ≤ 0.001). (**c**) Quantification of cells expressing EGFP plasmids (GFP+) that are concomitantly devoid of PURO incorporation [PURO(−)] from the experiment shown in (**a**). Error bars represent mean values ± SEM (*N* = 3, Two-way ANOVA; *P ≤ 0.05; **P ≤ 0.01; ***P ≤ 0.001).

To further evaluate the role of SG hyper-assembly in the translation defect caused by cancer-associated DDX3X mutations we took advantage of two RNA-binding proteins that are essential to SG assembly, G3BP1 and G3BP2^57^. As expected, concomitant depletion of endogenous G3BP1 and G3BP2 in HeLa cells prevented arsenite-induced SG assembly as monitored by immunostaining against the SG marker eIF4G (Supplementary Figure S9). We next assessed how inhibition of SG assembly impacted the translation defect associated with mutant DDX3X. We determined that depletion of endogenous G3BP1/G3BP2 significantly diminished MB-associated mutant DDX3X-induced SG formation (Fig. 8a,b, and Supplementary Figure S9), and, remarkably, also reversed the global translation defect (Fig. 8a,c). These results confirm that SG hyper-assembly is largely responsible for the global translation defect caused by cancer-associated DDX3X mutations and points towards a strategy of therapeutic intervention for the downstream consequences of DDX3X mutations.

## Discussion

The DEAD-box RNA helicase DDX3X has recently emerged a protein commonly mutated in multiple tumor types^2^,^3^,^4^,^5^,^6^,^7^,^8^,^9^,^10^, and it is among the most frequently mutated genes in pediatric and adult MB^11^,^12^,^13^,^14^,^15^. We undertook a comprehensive, unbiased evaluation of DDX3X function and the consequences of DDX3X missense mutations in cancer by analyzing a spectrum of these mutations occurring in MB tumors using cell biological, biochemical, genomic, and proteomic approaches. We found that cancer-related DDX3X mutations were associated with hyper-assembly of SGs that was accompanied by a striking impairment in mRNA translation (Figs 1 and 2). We performed a comprehensive assessment of the RNA targets bound by endogenous DDX3X, which revealed that DDX3X bound a specific pool of mature mRNAs with a particular enrichment toward 5′-UTRs and supported their translation (Figs 3 and 4). We also determined that cancer-associated variants did not substantially impact the ability of DDX3X to bind mRNA targets, although translation of target mRNAs was significantly impaired (Fig. 5). The significant reduction in global protein synthesis associated with expression of mutant DDX3X seemed greater than could be accounted for by the specific pool of DDX3X target mRNAs (Fig. 2). Thus, we examined global translation directly by ribosome profiling, a technique which provides a snapshot of ribosome occupancy across the breadth of the translatome^52^. This analysis indicated that expression of a MB-associated DDX3X mutant resulted in a global decrease of ribosome occupancy at coding sequences of mRNAs, with a concomitant accumulation of ribosomes at 5′-UTRs, consistent with a defect in translation initiation (Fig. 6). Remarkably, this analysis revealed that translation impairment was not limited to DDX3X targets, but also extended to mRNAs not bound by DDX3X (Fig. 6), indicating that MB-associated mutations in DDX3X cause a global impairment in translation. This broad impairment of translation in cells expressing mutant DDX3X as shown by ribosome profiling correlates with mutation-dependent hyper-assembly of SGs (Fig. 1). Indeed, a puromycin incorporation assay that permits cell-by-cell assessment of translation showed strong correlation between mutant DDX3X-dependent SG assembly and profoundly impaired translation (Fig. 2).

We propose that cancer-associated missense mutations in DDX3X results in a global impairment of protein synthesis by (i) directly binding and blocking translation initiation of target mRNAs that leads to the nucleation of SGs and the sequestration of these targets into SGs, and (ii) indirectly promoting the recruitment of non-target mRNAs and essential translation factors to SGs. This model predicts that global translation impairment caused by expression of mutant DDX3X could be limited or potentially reversed by inhibiting SG assembly. We investigated this possibility first by deleting the N- and C-terminal LCD sequences within DDX3X that contribute to the concentration-dependent liquid-liquid phase separation that underlies SG assembly^35^,^39^,^40^,^41^,^42^. Second, we inhibited SG formation by genetic depletion of G3BP1/2 factors that are essential for SG assembly. Indeed, deletion of N-terminal low complexity domain of DDX3X or genetic depletion of G3BP1/2, significantly reversed DDX3X-mediated SG hyper-assembly and restored translation as assessed by monitoring puromycin incorporation in nascent proteins and assessment of global protein synthesis (Figs 7 and 8). Thus, our data suggest that the SG hyper-assembly mediated by MB-associated mutations in DDX3X contributes to global translation inhibition independent of a primary effect at the level of translation initiation. Importantly, the fact that cancer-associated mutant DDX3 forms drive hyper-assembly of SGs even under non-stress conditions strongly suggest that the mutant forms of the protein may possess gain of function alterations that somehow bestow stress granule nucleator activity. Together, these results suggest that drugs that limit SG assembly and/or promote stress granule disassembly might reverse translation defects caused by cancer-associated DDX3X mutations and potentially contribute treatment strategies for tumors that harbor DDX3X mutations.

### Mechanistic insights into DDX3X role in mRNA translation

CLIP-seq showed that DDX3X preferentially binds 5′-UTR proximal to the AUG start codon (Fig. 3); and coincidentally, ribosome profiling revealed that expression of a MB-associated mutant DDX3X led to a significant accumulation of ribosomes within 5′-UTRs (Fig. 6b). Thus, we speculate that DDX3X may play an important role during late events of translation initiation, perhaps by resolving secondary structures at regions proximal to AUG start sites on mRNA targets or by positioning competent monosomes at AUG start sites and priming translation elongation. However, additional experimental data must be obtained to support these conclusions. Another interestingly finding from our CLIP-seq data was that DDX3X not only bound 5′-UTR regions on mRNAs, but unexpectedly, there was substantial binding within coding sequences and 3′-UTRs (Fig. 3c,d). These observations suggest the possibility that DDX3X participates in additional steps of the mRNA metabolism, including translation elongation or mRNA decay. Nevertheless, we did not detect significant changes in translation elongation upon expression of MB-associated mutations in DDX3X (Fig. 6). It will be interesting to elucidate the biological significance of DDX3X binding in coding regions and 3′-UTRs in future studies.

### Contribution of cancer-associated missense mutations in DDX3X to the etiology of the disease

The high recurrence of somatic DDX3X mutations in numerous cancer types suggests that perturbation of translational regulation by DDX3X plays an important role in tumor development. While it is possible that DDX3X missense mutations contribute to tumorigenesis through inhibited expression of a specific tumor suppressor, an alternative possibility is that altered translation due to DDX3X mutations contributes to tumor development as an adaptive mechanism to metabolic stress. This notion is consistent with a growing body of evidence indicating that deregulation of translation is a common mechanism by which diverse oncogenic pathways promote cellular transformation and tumor development^58^. Deregulation of translation is presumed to confer growth or survival advantages to tumor cells, in many cases by augmenting protein synthesis^59^. However, in certain circumstances inhibition of translation can confer survival advantages to tumor cells, and is particularly important for metabolic adaptation during nutrient or oxygen deprivation^59^. An excellent example of translation-related metabolic adaptation is the recent report that eukaryotic elongation factor 2 kinase (eEF2K), which is activated by AMP-kinase (AMPK), confers survival of MB tumor cells under acute nutrient depletion by blocking translation elongation^60^. We propose that DDX3X mutations act similarly, inhibiting global translation to support metabolic adaptation of MB cells; and it achieves this by promoting hyper-assembly of SGs during metabolic adaptation. However, we cannot exclude the possibility that mutation-dependent SG formation plays a role in the regulation of specific signaling pathways, either by inhibiting specific mRNAs or sequestering key molecules in SGs, thus contributing to tumorigenesis. Either case, drugs that disassemble SGs may disarm cancer cells in MB patients. Our data is consistent with recent findings that demonstrate that SGs play critical roles during tumor progression by promoting invasion and metastasis *in vivo*^61^. It is worth mentioning that despite we observe DDX3X aggregates resembling SGs in MB and other tumor types (Fig. 1 and Supplementary Figure S1), it remains to be determined whether these structures reflect an accumulation of SGs. Elucidating the precise mechanism whereby induction of SG assembly and inhibition of translation by mutations in DDX3X contribute to cancer remains to be determined but is only growing in importance.

While in MB tumors nearly all DDX3X mutations are nonsynonymous single nucleotide variants that are capable of expressing stable DDX3X proteins harboring single amino acid substitutions (Fig. 1a), other tumor types such as various blood cancers often display other DNA changes leading to frameshifts, spliced variants, and chromosome rearrangements between the *DDX3X* and other loci^2^,^3^,^5^,^6^. Thus, elucidating the precise mechanism whereby these genomic variations in DDX3X interfere with the normal function of this helicase in each individual tumor type and how they contribute to tumorigenesis remains to be determined but is only of growing importance.

## Material and Methods

### Constructs

Full-length human *DDX3X* cDNA clone was purchased from Thermo Scientific (Accession BC011819, Clone ID 3617040). Point mutations on DDX3X (T275M, G302V, G325E, M370R) that resembled mutations found in patients with MB were made using the QuickChange II XL Site-Directed Mutagenesis kit (Agilent Technologies). DDX3X point mutants A222P, R351W, L353F, D354V, and P568P were kindly provided by Dr. Eric Enemark (SJCRH). N-terminal FLAG- or GFP-tagged DDX3X^WT^ and the respective mutants were PCR-generated and cloned into EcoRI and XhoI sites in the mammalian expression vector pCDNA 3.1. Plasmid pCDNA3.1 FLAG-tagged DDX3X^DQAD^, which directs the expression of the DDX3X mutant harboring a mutation in the DEAD motif, was constructed with an E348Q substitution by QuickChange II XL Site-Directed Mutagenesis method. The pCDNA3.1 FLAG-tagged DDX3X^ΔIVa^ construct, which directs the expression of DDX3X lacking motif IVa, was done by deleting HGDRSQRDRE472-481 residues of human DDX3X by using the overlap-extension PCR method. All plasmids were verified by restriction digestion and sequencing methods. HEK293T cells stably expressing FLAG-DDX3X^WT^ or FLAG-DDX3X^G325E^ were generated by lentiviral transduction of a pCLEG-Tet2-FLAG DDX3X plasmid. The pCLEG-Tet2-FLAG-DDX3X plasmid was constructed by releasing the dsRedEX2 from the pCLEG-Tet2-dsRedEx1 plasmid (Vector Development and Production, SJCRH) and inserting the FLAG-wild-type or the DDX3X mutant G325E into the EcoRI and NotI positions. HEK293T cells were transduced with the lentiviral plasmid, plated at 1 × 10^5^ cells per well in a 6-well dish in DMEM +10% fetal calf serum. Transduced cells were selected by sorting GFP+ (which is expressed by a separate EF1a promoter within the pCLEG-Tet2-FLAG-DDX3X plasmid) cells by flow cytometry. For EGFP-DDX3X^WT/G325E^-ΔLCD1, PCR amplification was carried out from amino acid 115 to 661 with stop codon. For delta For EGFP-DDX3X^WT/G325E^-ΔLCD2, PCR amplification was carried out from amino acid 1 to 581 with stop codon. Overlap PCR extension method was followed using Pfu Ultra Hotstart enzyme to create EGFP-DDX3X^WT/G325E^-ΔLCD1/ΔLCD2. These fragments were cloned into pCDNA 3.1 at BamH1 and Xho1 sites.

### ^35^S-Met/Cys metabolic labeling

HEK293T or LRLPs cells seeded on poly-L-lysine treated 60 mm plates (~50% confluent) were transfected with 10 μg FLAG-DDX3X plasmids for 48 h or 100 nmol control/DDX3X siRNA oligos for 48 h. Media was removed and cells were washed with pre-warmed 1× PBS twice. Cells were incubated for 30 min in labeling media (DMEM, Corning, cat. no. 17-204-CL) supplemented with dialyzed FBS (w/o glutamine and methionine). 100 μCi ^35^S-Met/Cys (EasyTag EXPRESS Protein Labeling Mix, Perkin Elmer, cat. no. NEG772) diluted in labeling media was then added to plates for the desired time points. After each time point, cells were washed with 1× cold PBS twice, and lyzed in NP-40 buffer on ice for 20 min. Lysates were spun down at 14,000 r.p.m. at 4 °C for 10 min. ^35^S-Met/Cys incorporation was measured using liquid scintillation counting.

### Puromycin incorporation assay

For Figs 2 and 7, HEK293T cells were transfected with with 0.25 μg/well (4-well glass slide, Millipore) of control plasmid, or with the indicated EGFP-DDX3X fusion constructs for 24 h using FuGENE 6 as indicated by the company. For Fig. 8, HeLa cells were first treated with ON-TARGETplus human G3BP1 and G3BP2 siRNAs (Thermo Scientific, cat. no. L-012099-00 or LU-015329-01-0002), or ON-TARGETplus non-targeting control pool (Thermo Scientific, cat. no. D-001810-10) to a final concentration of 100 nmol using Dharmafect 1 transfection reagent for 24 h as indicated by the company. Then, cells were transfected with EGFP plasmids as indicated above. After transfection, pulse labeling was done with Puromycin (InvivoGen, cat. no. ant-pr-1) diluted in DMEM media (1 μg/mL) for 30 min at 37 °C and with a 5% CO_2_ concentration. Cells were then washed with 1× PBS and immunofluorescence was performed next against Puromycin and eIF4G or G3BP1/2. Images were captured using a LSM510 (Zeiss) confocal microscope with a 63X or 20X objectives and Zeiss ZEN software.

### RNA-seq

For RNAseq analyses, DDX3X was knock-down by treating HEK293T cells with control or DDX3X siRNA oligos (for information of siRNA oligos refer to supplementary information) for 48 h, whereas for ectopic expression of DDX3X cells were transfected with 5 μg GFP alone or GFP-DDX3X variants for 32 h. GFP+ cells were then collected by flow cytometry (BD Biosciences). RNA-sequencing libraries were prepared using approximately 1 μg total RNA using TruSeq RNA Sample Prep v2 kits per the manufacturer’s instructions (Illumina). Details of statistical procedures are provided in supplemental information.

### CLIP-seq

See supplemental information for detailed CLIP-seq. Briefly, for CLIP-seq HEK293T cells were UV treated and then lysed with NP-40 buffer. Immunoprecipitation of endogenous DDX3X was performed using the mouse monoclonal anti-DDX3X antibody (clone 2253C5, Santa Cruz) in samples previously treated with DNAse and various concentrations of RNAse. RNA was labeled at the 5′ end with γP^32^ ATP by using T4 polynucleotide kinase. RNA/DDX3X complexes were resolved by SDS-PAGE and transferred to nitrocellulose membranes. RNA was then extracted, and the library was prepared. Details of statistical procedures are provided in supplemental information.

### Ribosome profiling

HEK293T cells plated at low confluency were transfected with either empty vector or FLAG-tagged DDX3X constructs for 24 h. To stall ribosomes to mRNA, cycloheximide (100 μg ml^−1^; Sigma-Aldrich) was added to cells for 5 min. Cell lysis, nuclease footprinting, ribosome recovery, and RNA-library preparation were done as previously described^52^. After the library construction, deep sequencing was performed as for CLIP-seq. Details of statistical procedures are provided in supplemental information.

## Additional Information

**Accession codes**: The Gene Expression Ommnibus code for CLIP-seq, RNA-seq, and Ribosome profiling is GSE59096 (subseries: GSE59093, GSE59094, and GSE59095).

**How to cite this article**: Valentin-Vega, Y. A. *et al.* Cancer-associated DDX3X mutations drive stress granule assembly and impair global translation. *Sci. Rep.* **6**, 25996; doi: 10.1038/srep25996 (2016).

## Supplementary Material

Supplementary Information

Supplementary Table S1

Supplementary Table S2
