# Author Correction: Cancer-associated DDX3X mutations drive stress granule assembly and impair global translation

**DOI:** 10.1038/s41598-024-63662-z

**Published:** 2024-06-06

**Authors:** Yasmine A. Valentin-Vega, Yong-Dong Wang, Matthew Parker, Deanna M. Patmore, Anderson Kanagaraj, Jennifer Moore, Michael Rusch, David Finkelstein, David W. Ellison, Richard J. Gilbertson, Jinghui Zhang, Hong Joo Kim, J. Paul Taylor

**Affiliations:** 1 Department of Cell and Molecular Biology, St. Jude Children’s Research Hospital, Memphis, TN 38105 USA; 2 Department of Computational Biology, St. Jude Children’s Research Hospital, Memphis, TN 38105 USA; 3 Department of Oncology, Cambridge Cancer Centre, Cancer Research UK Cambridge Institute, Cambridge, UK; 4 Department of Pathology, St. Jude Children’s Research Hospital, Memphis, TN 38105 USA; 5 Howard Hughes Medical Institute, Chevy Chase, MD 20815 USA

Correction to: *Scientific Reports* 10.1038/srep25996, published online 16 May 2016

This Article contains errors.

Figure 2a, Figure 5d and Figure 5e represent different modes of interrogation of the same samples from one experiment. Figures 2a and 5d should have shown the same western blotting data for FLAG-DDDX3X and β-Actin, but the samples from different time points have been incorrectly labelled as biological replicates in Figure 5d. Additionally, the mutant M370R has been mislabelled as G302V in both Figure 5d and Figure 5e. The corrected Figure 5 and accompanying figure legend are included below. These changes do not affect the conclusions of the article.

**Figure 5:**
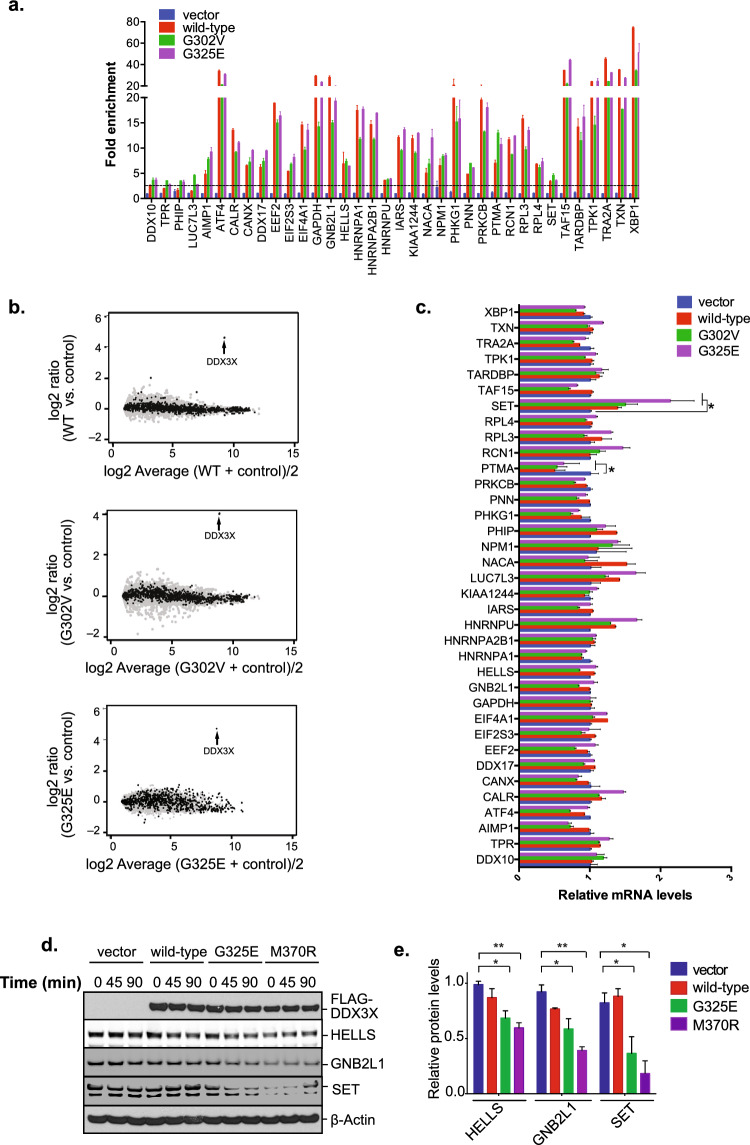
MB-associated mutations in DDX3X impact the translation of mRNA targets. (**a**) RNA immunoprecipitation analysis of numerous DDX3X targets using exogenous FLAG-tagged wild- type DDX3X and two cancer mutants (G302V and G325E). Two non-target RNAs were analyzed as negative control (DDX10 and TPR). Cells transfected with empty vector and immunoprecipitated with the anti-FLAG antibody M2 served as control. 87.5% of DDX3X mRNA targets were validated by the assay (horizontal black-dotted line represents cut-off based on non-target RNAs). (**b**) MA plot comparing RNA-seq data sets from HEK293T cells transfected with empty vector or a DDX3X-expressing vector (wild-type and two mutants: G302V and G325E). Transfections efficiencies are confirmed by the detection of high mRNA levels of DDX3X itself (denoted by the black arrows). Black dots represent DDX3X targets, gray dots represent non-targets. (**c**) Reverse transcription–qPCR analysis of several DDX3X CLIP-seq targets plus two non-targets (DDX10 and TPR) in HEK293T cells in cells expressing either wild- type DDX3X or two cancer-related mutant (G302V and G325E). Mean ± SEM values are based on a minimum of two replicated experiments. ~91% of DDX3X mRNA targets showed insignificant changes in their levels between cells expressing vector and those expressing DDX3X variants (Multifactorial ANOVA: *P ≤ 0.01). (**d**) Western blot analyses in HEK293T cells transfected with FLAG-tagged wild-type DDX3X or two cancer-related mutants (G325E and M370R). The experimental samples used for global translation analysis in Fig. 2a were interrogated in parallel by immunoblotting for CLIP-seq targets HELLS, GNB2L1 and SET. This approach demonstrated that expression of cancer-related DDX3X mutants, but not wild-type DDX3X, reduces steady-state levels of proteins produced by DDX3X target mRNAs. Indicated timepoints correspond to 35S labeling as assessed in Fig 2a. Whereas Fig. 2a uses defined timepoints to assess the effects of DDX3X mutations on global translation, (**d**) interrogates the effects of these mutations on the steady-state levels of specific targets. The FLAG and β-Actin blots are reproduced here for convenience. (**e**) The mean protein levels of three timepoints of samples shown in (d) was graphed. Error bars represent mean values ± SEM (Student’s t-test between control or cells expressing DDX3X variants; *P ≤ 0.05; **P ≤ 0.01).

