# Supplementary material for: Cancer-associated DDX3X mutations drive stress granule assembly and impair global translation
Source: Sci Rep. 2016 May 16;6:25996. doi: 10.1038/srep25996 (PMC4867597; doi:10.1038/srep25996)
Supplement: Supplementary Information [file srep25996-s1.pdf]

SUPPLEMENTARY INFORMATION

Cancer-associated DDX3X mutations drive stress granule assembly and impair global translation

Yasmine A. Valentin-Vega[1], Yong-Dong Wang[2], Matthew Parker[2], Deanna M. Patmore[3], Anderson Kanagaraj[1], Jennifer Moore[1], Michael Rusch[2], David Finkelstein[2], David W. Ellison[4], Richard J. Gilbertson[3], Jinghui Zhang[2], Hong Joo Kim[1], J. Paul Taylor[1,5*]

[1]Department of Cell and Molecular Biology, St. Jude Children's Research Hospital, Memphis, TN 38105, USA

[2]Department of Computational Biology, St. Jude Children's Research Hospital, Memphis, TN 38105, USA

[3]Department of Oncology, Cambridge Cancer Centre, Cancer Research UK Cambridge Institute, Cambridge, UK

[4]Department of Pathology, St. Jude Children's Research Hospital, Memphis, TN 38105, USA

[5]Howard Hughes Medical Institute, Chevy Chase, MD 20815, USA

[*]Correspondence: jpaul.taylor@stjude.org

Keywords: DDX3X, Belle, stress granules, translation impairment, CLIP-seq, ribosome profiling, mRNA, medulloblastoma, G3BP1, G3BP2.

Short Title: Cancer-related DDX3X mutations impair translation

Supplemental figure legends:

Supplemental figure S1. High expression and punctated distribution of DDX3X in human cancer.

a. Histopathological assessment of DDX3X expression in a panel of pediatric medulloblastomas and normal brain tissues. Representative images obtained from normal brain cortex and cerebellum and 10 pediatric medulloblastomas. The scores for relative protein level among the samples are presented (see Material and Methods). Three MB tumors carrying mutations DDX3X (G302V, M370R, and G325E) and various tumors carrying the wild type form show high levels and punctated distribution of the protein. Some panels presented here are duplicated from Figure 1b (refer to text). *WT*, wild-type DDX3X. Scale bar: 20 µm.
b. High levels and punctated distribution of DDX3X protein are observed in numerous human tumors. Immunohistochemistry analysis of normal human tissues (*left panels*: brain cortex, cerebellum, skin and breast) and three common human tumors (*right panels*: malignant glioma, melanoma, and breast carcimoma) retrieved from The Human Protein Atlas public database (www.proteinatlas.org). High DDX3X protein levels and punctated distribution of DDX3X are observed in the cytoplasm of tumor cells (right panels). Arrows point to DDX3X aggregates. Low levels of DDX3X are observed in normal tissues.

Supplemental figure S2. MB-associated mutations in DDX3X induce SG formation.

a. Immunofluorescence against endogenous DDX3X and the SG marker eIF4G in HeLa (left panel) and LRLPs (right panel) cells left untreated or under heat shock conditions (43 °C, 1 h). For right panels (LRLPs cells), green color represents endogenous DDX3X staining while red color represents eIF4G. Nuclei were stained with DAPI.
b. HeLa cells were transiently transfected with FLAG-tagged wild-type DDX3X or various cancer-related mutants for 24 h followed by Western blot analysis using anti-FLAG (top panel), anti-DDX3X (middle panel), or β-actin (bottom panel). The data shown is representative of the relative protein levels that are observed among the DDX3X variants obtained by transient transfection in various cells lines (FLAG immunoblotting). The relative levels between exogenous and endogenous DDX3X proteins are shown by immunoblotting with an anti-DDX3X antibody. Similar results are obtained with EGFP-tagged proteins (data not shown).
c. *Tet*-On inducible stable HEK293T cells expressing FLAG-tagged wild-type DDX3X or the MB-associated mutant G325E were treated with doxycycline (DOX) for the indicated time and the induction of DDX3X was monitored by immunoblotting using either anti-FLAG (top panels) or anti-DDX3X (bottom panels) antibodies. β-actin served as the loading control.
d. *Tet*-On inducible HEK293T cells (*c*) were treated with 1 μg mL^{-1} doxycycline for 24 h and then, cells were left untreated or incubated at 43 ºC for 1 h to induce heat shock. Then, immunostained was performed against FLAG to detect DDX3X (green) or eIF4G (red). Boxes denote the magnified region at the right. Scale bars are shown.

e. Quantification of DDX3X granules in the experiment shown in (*d*). Mean ± SEM values are based on a minimum of three replicated experiments. Student *t*-test is shown.

f. Pulse chase experiment performed in the *Tet*-On inducible HEK293T cells expressing either FLAG-tagged wild-type DDX3X or the G325E DDX3X mutant Tet-On inducible HEK293T cells were treated with 1 µg mL^{-1} doxycycline for 24 h (pulse), washed with PBS and maintained in normal media for the indicated time points (chase) (see Materials and Methods). Western blot was performed against FLAG-DDX3X and β-actin. % residual DDX3X is shown for each sample. Mutant DDX3X showed comparable (or even less) stability thank wild-type DDX3X.

Supplemental figure S3. MB-associated DDX3X mutants are capable of binding RNA *in vivo.*

a. *In vivo* RNA binding assay of wild type DDX3X, three cancer related mutants (G302V, G325E, and M370R), enzymaticatically defective DQAD, and RNA-binding deletion (motif IVa). Shown are the DDX3X/RNA complexes detected by chemiluminescence after 3' end labeling of RNA as described in Material and Methods.

b. Western blot analyses of immunoprecipitated material from the RNA binding assay shown in (*a*). DDX3X was detected using anti-FLAG antibody.

c. Bar graph showing the relative RNA binding capabilities of DDX3X variants measured by calculating the ratio between RNA intensity and the DDX3X protein levels shown in (*a-b*). The experiment is representative of two independent experiments.

Supplemental figure S4. CLIP-seq analysis of endogenous DDX3X in HEK293T cells.

a. Various commercially available antibodies were used to immunoprecipitate endogenous DDX3X in HEK293T cells. Subsequently, Western blot analyses were performed against DDX3X using an antibody from Bethyl Laboratories (A300-474A). Mouse monoclonal antibody 2253C5a (Santa Cruz Biotechnologies) proved to be most efficient among all antibodies tested for immunoprecipitation analysis (denoted by a red square).
b. Immunoprecipitated material using the mouse monoclonal antibody 2253C5a shown in (*a*) was resolved by SDS-PAGE and stained with Sypro-ruby (not shown). Band corresponding to the expected size for DDX3X was excised from the gel and analyzed by mass-spectrometry, identifying DDX3X. Shown in yellow color are the unique peptides corresponding to DDX3X identified by mass spectrometry.
c. Table summarizing the number of reads obtained for two independent DDX3X CLIP-seq experiments (CLIP-A and CLIP-B) and IgG control.
d. Logarithmic plots of the number of uniquely mapped reads per gene for the CLIP A and CLIP B experiments. Pearson correlation coefficients indicate comparison of CLIP-seq experiments. Each dot represents a gene.
e. Examples of DDX3X targets identified by CLIP-seq. CLIP-seq reads mapped to hnRNPA1, EEFA1, EIF4A1, and GNB2L1 RNAs are shown. 5’UTR, coding sequences, and 3’UTR regions are denoted in green, gray, and orange, respectively.

Supplemental figure S5. Ribosome profiling in HEK293T cells expressing vector, wild-type or MB-associated mutant G325E DDX3X forms.

a. Table summarizing the the raw reads and the reads uniquely mapped to the human genome in ribosome profiling experiments. Ligation control represents a defined oligo submitted for library preparation equivalent to the experimental groups.

b. The ribosomal densities of three representative mRNAs showing high densities upstream of start codons and concomitant decreases in coding regions. Red color shows ribosomal densities upstream of start codons; blue color shows densities in the coding sequences. Organization of mRNA structures (minus introns), are denoted at the bottom in gray color (thick lines, coding sequences; thin lines, UTR regions).

Supplemental figure S6. Removing the N-tarminal LCD prevents DDX3X from inducing SG formation.

a. HeLa cells transfected with the EGFP control expressing plasmid or plasmids expressing the indicated EGFP-tagged DDX3X variants for 24 hr. Then, cells were treated with 0.5 mM sodium arsenite for 30 min followed by fixation and immunostaining against G3BP. Note that removing the ΔLCD1 of DDX3X prevents SG formation in a fraction of HeLa cells (white arrows). Green represents EGFP signal, magenta represents endogenous G3BP. Nuclei were visualized by staining with DAPI. Scale bars are shown.

b. Quantification of SGs in HeLa cells from experiment shown in panel (*a*). Mean ± SEM values are based on a minimum of three biological replicates. Student's *t*-test comparison between full-length DDX3X and either ΔLCD1 or ΔLCD2 mutants is shown (*, $P \leq 0.05$).

Supplemental figure S7. Treatment with ISRIB does not prevent cancer-associated mutant DDX3X from inducing SGs.

a. HeLa cells were pre-treated for 8 h with DMSO or 200 nM ISRIB and then transfected with EGFP alone (not shown) or the indicated EGFP-tagged DDX3X plasmids for 24 h (DMSO or ISRIB were kept during the transfection period). Then, cells were left untreated or co-treated with 0.25 mM sodium arsenite for 30 min. Immunofluorescence was performed next against G3BP1 (red). EGFP was used to visualize DDX3X. White arrows depict HeLa cells treated with ISRIB displaying SGs as monitored by G3BP1 and DDX3X positivity. Note that under normal culture conditions (left panels), ISRIB treatment did not prevent DDX3X from inducing SGs. During oxidative stress, ISRIB partially prevented SG formation in HeLa cells (right panels; white-dashed lines), but did not effectively prevent SG formation in cells expressing DDX3X (right panels, white arrows). Scale bars: 20 µm.
b. Quantification of HeLa cells treated with 200 nM ISRIB or co-treated with 200 nM ISRIB and 0.25 mM sodium arsenite. Note that ISRIB treatment prevented SG formation only partially in HeLa cells (~ 30% ISRIB-treated cells lacked SGs as monitored by G3BP1 staining).
c. Quantification of SGs in HeLa cells treated with DMSO or ISRIB during normal culture conditions from experiment shown in *(a)* (*left panels*). SGs were monitored using the SG marker G3BP1 (red). N=3, Student *t* test between cells treated with DMSO and ISRIB.
d. Quantification of SGs in HeLa cells co-treated with sodium arsenite and ISRIB (or DMSO) from experiment shown in *(a)* (*right panels*). SGs were monitored

using the SG marker G3BP1 (red). Note that the reduction in SG formation in HeLa cells expressing DDX3X during stress conditions is comparable to untransfected HeLa cells (compare panels *b* and *d;* ~30% of total cell population lack SGs regardless of DDX3X expression). N=3, ***; $P < 0.001$, Student *t* test between cells treated with DMSO and ISRIB.

Supplemental figure S8. Treatment with the PERK inhibitor GSK2606414 does not prevent cancer-related mutant DDX3X from inducing SG formation.
HeLa cells were pre-treated with DMSO or 500 nM PERK inhibitor (GSK2606414) for 8 hr. Then, cells were transfected with the indicated EGFP-tagged DDX3X plasmids in the presence of the inhibitor or DMSO. 24 h after transfection, cells were then fixed and immunofluorescence was performed against the SG marker eIF4G (red). Arrows denote cells displaying DDX3X-induced SGs. Green indicates EGFP signal; nuclei were stained with DAPI. Scale bars: 20 µm. The experiment was performed three times.

Supplemental figure S9. Knock-down of G3BP1/G3BP2 prevents MB-associated DDX3X mutant from inducing SG formation during stress conditions.

a. Proof-of-principle experiment demonstrating a failure in SG assembly mediated by knocking-down the SG nucleator factors G3BP1 and G3BP2. HeLa cells were treated with control or G3BP1/G3BP2 siRNAs for 24 h, and then transfected with the indicated EGFP-tagged DDX3X plasmids for another 24 h. Cells were then treated with 0.5 mM sodium arsenite for 30 min followed by fixation and immunofluorescence analysis against the SG markers G3BP1 (red) and eIF4G (magenta). DDX3X expression pattern is traced with EGFP. Nuclei are stained with DAPI.

b. Quantification of cells with DDX3X in SG after arsenite treatment from experiment shown in *(a).* The percentage of EGFP+ cells that show relocalization of EGFP-tagged DDX3X variants to SGs after arsenite treatment was graphed. Nearly 100% of cells transfected with EGFP-tagged DDX3X showed DDX3X relocalization to SG under stress condition when control siRNA oligos were used; whereas only ~30% of cells treated with G3BP1/G3BP2 siRNAs showed DDX3X in SG. (N=3, ***; $P < 0.001$, Student *t* test between si-control and siG3BP1/siG3BP2 treated samples).

Supplementary Table S1. List of peaks identified in the DDX3X CLIP-seq experiments The table presents all the high confident peaks identified in the DDX3X CLIP-seq experiments (CLIP-A and CLIP-B) (see Material and Methods).

Supplementary Table S2. Gene Ontology analysis of DDX3X CLIP-seq targets David Bioinformatic Resources 6.7 was used to perform gene ontology analyses. mRNAs encoding proteins involved in translation are enriched among the DDX3X mRNA targets.

SUPPLEMENTAL MATERIAL AND METHODS

Antibodies

The following antibodies were used in this study: anti-DDX3X (mouse monoclonal 2253C5a, Santa Cruz Biotechnologies, sc-81247; rabbit polyclonal, Bethyl, cat. no. A300-475A; rabbit polyclonal, Bethyl, cat. no. A300-474A; rabbit polyclonal Q-21, Santa Cruz Biotechnologies cat. no. sc-130736; rabbit polyclonal, Millipore, cat. no. 50-172-797), anti-eIF4G (H-300, Santa Cruz Biotechnologies, cat. no. sc-11373), anti-Puromycin (3RH11, KeraFAST, cat no. EQ0001) anti-DDX17 (S-17, Santa Cruz Biotechnologies, cat. no. sc-86409), anti-FLAG (M2 cone, Sigma, cat. no. F1804), anti-β Actin (clone, AC-15, Sigma, cat. no. A1978), anti-SET (Abcam, cat. no. ab1183), anti-HELLS (Cell Signaling, cat. no. 7998S), anti-GNB2L1/RACK1 (B-3, Santa Cruz, cat. no. sc-17754), anti-hnRNPA2B1 (EF-67, cat. no. sc-53531), anti-PNN (4FQ, Santa Cruz Biotechnologies, sc-101127), anti-GFP (Santa Cruz Biotechnologies, cat. no. sc-8334), anti G3BP1 (BD transduction, cat. no. 611126; Santa Cruz Biotechnology, H-94, sc-98561).

Cell culture, transfections and treatments

HEK293T or HeLa cells were grown in Dulbecco's Modified Eagle's Serum, DMEM (Hyclone, cat. no. SH30243.01), supplemented with 10% fetal bovine serum, FBS (Hyclone, cat. no. SH30079.02) and 1% Pen-Strep (100 U/ml penicillin, 100 µg/ml streptomycin; Gibco, cat. no. 15140-122). Lower Rhombic lip cells (LRLPs) were maintained as described [1]. Cells were incubated at 37 °C and with a 5% CO_2 concentration. For Western blot assessments in HEK293T cells, 8-16 h prior transfection cells were seeded in 60 mm or 10 cm dishes to obtain a confluency of ~ 80% the time of transfection. Cells were then transfected with 2-5 µg plasmids using

Lipofectamine 2000 (Life Technologies) according to the manufacturer's instructions. For fluorescence studies, 16 h prior to transfection, cells were plated onto 4-well chamber slides so that at the time of transfection cells were 70% confluent. Cells were transiently transfected using FuGENE 6 (Promega, cat. no. E2691) as indicated by the manufacturer's instructions. To induce cellular stress, cells were incubated at 43 °C for 1 h or with 0.5 mM sodium arsenite for 30 min (Sigma, cat. no. 35000) 24 h after transfection. For siRNA treatment, ON-TARGETplus human or mouse DDX3X siRNAs (Thermo Scientific, cat. no. L-006874-00 or L-043597-00), and ON-TARGETplus non-targeting control pool (Thermo Scientific, cat. no. D-001810-10) were transfected into respective cells to a final concentration of 100 nmol per 60 mm culture plate using Dharmafect 1 transfection reagent for 48 h as indicated by the company (Thermo Scientific, cat. no. T-2001).

Cell immunostaining, stress granule counting, and microscopy

Cells were washed with phosphate-buffered saline (PBS, Hyclone, SH30028.02) and fixed with a 1:1 solution of acetone and methanol on ice, permeabilized with 0.1% Triton X-100 (Thermo Scientific, R21902) in PBS-T (0.1% Tween-20—Thermo Scientific, cat. no. 28321— in PBS) and blocked with 5% normal horse serum (Vector, cat. no. S-2000) in PBS-T, both at room temperature. Samples were incubated with primary antibodies at a 1:200 concentration in blocking solution overnight at 4 °C. Samples were washed three times with PBS-T, and secondary antibodies were added for 1 h at room temperature at 1:5000 final concentration in blocking solution. Cells were washed three times with PBS-T and coverslips were mounted with VECTASHIELD HardSet mounting media with DAPI. Images were captured using a LSM510 (Zeiss) confocal microscope with a 63X or 20X objectives and Zeiss ZEN

software. Scale bars are presented in each image panel. Minimal imaging processing (brightness/contrast and cropping) was performed using Adobe Photoshop CS6 or Image J64 softwares according to the acceptable imaging policies.

To stain for DDX3X and stress granule localization we used mouse monoclonal α-FLAG for FLAG-DDX3X and rabbit polyclonal α-eIF4G. GFP-tagged proteins were used in some experiments to visualize DDX3X. To quantify SGs, cells were scored for presence of FLAG- (or GFP-) tagged DDX3X and whether or not exogenous DDX3X-positive SGs were present. At least 200 cells were counted per sample and each sample was repeated three times.

Chemicals

ISRIB compound was purchased from Sigma (# SML0843). GSK2606414 compound was purchased from Millipore (#516535). For experiments shown in Supplemental figure 7, HeLa cells were pre-treated with DMSO or 200 nM ISRIB for 8 h and then transfected with EGFP alone or the indicated EGFP-tagged DDX3X plasmids for 24 h (DMSO or ISRIB were kept during the transfection period). Then, cells were left untreated or co-treated with 0.25 mM sodium arsenite for 30 min. Immunofluorescence was performed next against G3BP1. For experiments shown in Supplemental figure 8, HeLa cells were pre-treated with DMSO or 500 nM PERK inhibitor (GSK2606414) for 8 hr. Then, cells were transfected with the indicated EGFP-tagged DDX3X plasmids in the presence of the inhibitor or DMSO. 24 h after transfection, cells were then fixed and immunofluorescence was performed against the SG marker eIF4G.

Western blot

For cell lysis, NP-40 lysis buffer (50mM Tris HCL, 150mM NaCl, 0.5% NP-40, 50mM NaF) was added and samples incubated for 10 min on ice. Samples were centrifuged

for 10 min. at 4 °C, 14,000 r.p.m., and the supernatant removed. Soluble protein levels were quantified using a Bradford protein assay kit (Bio-Rad, cat. no. 500-0205). Samples were denatured by boiling for 3 min in 4X NUPAGE LDL sample buffer (Life Technologies, cat. no. NP0007) supplemented with β-mercaptoethanol and were run in a 4-12% NUPAGE Bis-Tris gel (Invitrogen). Protein were transferred into a nitrocellulose membrane (Amersham, cat. no. RPN3032D) using a semi-dry system at 13 V for 1 h, and membranes were blocked with 5% milk in PBS-T for 30 min. Membranes were incubated overnight in primary antibodies (1:1000 dilution in blocking solution). Secondary antibodies (either IgG HRP-conjugated antibodies from Amersham or dye-labeled secondary antibodies from LI-COR) were used at 1:10000 dilution in blocking solution for 1 h. For HRP-conjugated antibodies, proteins were detected by ECL (Amersham, cat. no. RNP2106) or Supersignal West Dura (Thermo Scientific, cat. no. 34076). Image J64 was used to quantify bands detected by western blot for some experiments.

Pulse chase

Tet-On inducible HEK293T cells were treated with 1 μg mL^{-1} doxycycline for 24 h (pulse) and then washed with PBS three times. Cells maintained in normal media without doxycycline for the indicated time points (chase). Protein lysates were obtained and Western blot was performed against FLAG-DDX3X and β-actin. β-actin was used to normalyzed relative DDX3X levels in each sample, and the residual DDX3X was calculated by designing DDX3X levels in time point 0 min as 100%. Image J64 was used to quantify the relative levels of DDX3X.

Analysis of human medulloblastomas

Human tumor samples were obtained with informed consent through an institutional review board approved protocol at SJCRH. For immunofluorescence, tissue microarrays of human tumor samples were stained using standard protocol in association with anti-DDX3X antibody (Bethyl, cat. no. A300-475A, 1:200), and nuclei were counterstained with 4',6-diamidino-2-phenylindole (DAPI; Vector Labs). Images were captured using a LSM510 (Zeiss) confocal microscope with a 63X objective and Zeiss ZEN software. Protein expression and punctate intensity were measured using the ImageJ64 software (ImageJ 1.47v). All images were analyzed using the same threshold and the image particles were then analyzed. The average number of particles was used for the punctate intensity. The protein expression was calculated by measuring the intensity after setting a common threshold for all images.

CLIP-seq

HEK293T cells were cross-linked on ice with UV radiation (254 nm) once at 400 mJ per cm^2 followed by a second UV treatment using 200 mJ per cm^2 in the presence of cold PBS (5 ml per 10 cm dish). Cells were pelleted at 1,000 rpm at 4 °C and stored at –80 °C until further use. Cells lysis was performed in cold Nonidet P-40 buffer (50mM Tris-HCl, pH 7.5, 150mM NaCl, 0.5% Nonidet P-40, 50mM NaF, and 1 × proteinase inhibitor cocktail; Roche Diagnostics, cat. no. 11836145001) for 10 min on ice followed by addition of a 1:100 volume of RQ1 DNase (Promega, cat. no. M6101) and incubation at 37 °C for 5 min at 1000 r.p.m. in Thermomixer (Eppendorf). After DNAse treatment, RNAse I was added (0.0–2 U, Life Technologies, cat. no. AM2294) and samples were incubated for 4 min at 37 °C with 1000 r.p.m. agitation in Thermomixer. Protein cell extracts were centrifuged for 10 min at 14,000 r.p.m. at 4 °C. For immunoprecipitation, the supernatant was incubated at 4 °C for 3.5 h with 10 µg of anti-DDX3X antibody (mouse monoclonal 2253C5a) or mouse IgG (Santa Cruz

Biotechnologies, cat. no. sc-2025) previously conjugated to 50 μl protein G Dynabeads (Life Technologies, cat. no. 10004D). Beads were washed three times with 500 μl cold NP-40 lysis buffer, twice with 500 μl high salt buffer (5X PBS, 0.5% NP-40), twice with 500 μl PNK buffer (50 mM Tris-HCl, pH 7.5, 0.5% NP-40 and 5 mM $MgCl_2$), and once with 500 μl nuclease-free water (Life Technologies, cat. no. AM9932). The beads were re-suspended in 40 μl nuclease-free water and treated with Calf Intestinal Alkaline Phosphatase as indicated by the company (Promega, cat no. M182A). Beads were washed twice with PNK+EGTA buffer (50 mM Tris-HCl, pH 7.5, 0.5% NP-40, 20 mM EGTA), and twice with PNK buffer. 5' RNA labeling was done by treating samples with 1 μl PNK enzyme (10 units; NEB), 1× PNK B buffer (NEB) and 1 μl $\gamma^{32}P$ ATP (10 mCi ml^{-1}, 3,000 Ci/mmol; Perkin-Elmer). After 10 min incubation at 37 °C at 1000 r.p.m., 10 μl 1mM ATP was added to beads and incubation was continued for 5 min. Beads were washed two times with 500 μl high salt buffer and three times with with 500 μl PNK buffer. RNA/DDX3X complexes were eluted with 50 μl 4X NUPAGE LDL sample buffer (Life Technologies, cat. no. NP0007) supplemented with β-mercaptoethanol by mixing for 10 min at 1,000 r.p.m. at 70 °C, and complexes were resolved in a 4-12% NUPAGE Bis-Tris gel (Life Technologies). Complexes were transferred into a nitrocellulose membrane (Life Technologies, cat. no. LC-2001). For RNA isolation, nitrocellulose membranes were fragmented with a clean scalpel blade and incubated with proteinase K solution (4 mg/ml proteinase K, 0.1 M Tris-HCl, pH 7.5, 10 mM EDTA and 50 mM NaCl) for 20 min at 37 °C with 1,000 r.p.m. agitation. The proteinase K treatment was repeated in the same buffer supplemented with 7 M urea before RNA extraction using Acid Phenol:Chloroform (Life Technologies, cat. no. AM9722) and ethanol precipitation. Complementary DNA (cDNA) libraries were prepared with the Nextflex Small RNA Sequencing Kit according to the manufacturer's instructions (Bioo-Scientifics, cat. no. 5132-01). For the library preparation a microRNA provided by the

company, which does not match any known sequence in miBase was used as control (5'Phos/CUCAGGAUGGCGGAGCGGUCU/3'). Sequencing was performed on an Illumina HiSeq 2000 using TruSeq SBS v3 reagents according to the manufacturer's instructions.

RNA binding assay

After transfection with the desired plasmids for 32 h in 10 cm dish, HEK293T cells were UV-treated and collected as for CLIP-seq experiment. Cell lysis was done using Nonidet P-40 buffer (CLIP-seq protocol) for 10 min at 4 °C. DNAse treatment (RQ1 DNAse, Promega) was done for 5 min at 37 °C followed by RNAse I treatment for 3 min at 37 °C (1U, Life Technologies). Supernatant was collected after centrifugation (10 min, 14,000 r.p.m, 4 °C). Immunoprecipitation was done as for CLIP-seq experiment using 5 µg FLAG M2 antibody. Immunoprecipitated DDX3X/RNA complexes were washed two times with Nonidet P-40 buffer, two times with high salt buffer, and two times with PNK buffer. RNA labeling was done on beads using the RNA 3' End Biotynilation Kit as indicated by the company (Pierce, cat. no. 20160). DDX3X/RNA complexes were eluted with 50 µL 4X NUPAGE LDL sample buffer, ran in 4-12% NUPAGE Bis-Tris gel, and transfered to nitrocellulose membrane as in CLIP-seq experiment. RNA detection was done using the Chemiluminescence Nucleic Acid Detection Kit as indicated by the company (Pierce, cat. no. 89880).

RNA immunoprecipitation (RIP) and RT-qPCR

For experiments using exogenous DDX3X variants, HEK293T were transfected with 5 µg pCDNA3.1 FLAG-tagged DDX3X plasmids in 10 cm culture plates for 32 h. Plates were washed once with 1× cold PBS and treated once with 400 mJ per cm^2 UV

followed by a second UV treatment using 200 mJ per cm^2 in the presence of cold PBS (5 ml per 10 cm dish). Cells were collected by centrifugation for 5 min at 2000 r.p.m. at 4 °C, resuspended in 1 mL PBS and centrifuged again at maximum speed for 10 seconds. Pellet were stored at –80 °C until further use. Cells lysis was performed in cold Nonidet P-40 buffer for 10 min on ice followed by addition of a 1:50 volume of RQ1 DNase and incubation at 37 °C for 10 min at 1000 r.p.m. Protein cell extracts were centrifuged for 10 min at 14,000 r.p.m. at 4 °C. The supernatant was incubated at 4 °C for 3.5 h with 10 µg of anti-FLAG M2 antibody previously conjugated to 50 µl protein G Dynabeads. Beads were washed three times with 500 µl cold NP-40 lysis buffer, twice with 500 µl high salt buffer, twice with 500 µl PNK buffer. For RNA isolation, beads were treated with proteinase K as in CLIP-seq experiment. RNA extraction was done using Acid Phenol:Chloroform and ethanol precipitation. One-step RT-qPCR was performed using TaqMan RNA-to-Ct 1-Step kit according to the manufacture's instructions (Applied Byosystems, cat. no. 4392938) in costume designed Taqman array fast plates containing primer/probe sets against DDX3X targets (Applied Biosystems). RT-qPCR for input RNA was also done using 5% of each sample. For mRNA expression analyses, total RNA was extracted from HEK293T cells (previously transfected with FLAG-DDX3X constructs for 32 h or with control/DDX3X siRNA oligos for 48 h) using RNeasy kit according to manufacturer's instructions (Qiagen) followed by one-step RT-qPCR analysis as indicated above. Quantitative PCR analyses were done using a 7900HT Fast Real Time PCR System (Applied Biosystems, SDS Software 2.2).

Ribosome half-transit time measurements

HEK293T cells (~ 50% confluent) were transfected in 15 cm dish with 15 µg plasmids for 24 h using Lipofectamine 2000. After transfection, cells were trypsinzed (TripLE

Express, GIBCO) and washed with 5 mL 1× PBS. Cells were then incubated with 5 mL labeling media for 30 min (see above). ^{35}S-Met/Cys was then added to a final dilution of 50 µCi per mL. At the indicated time points, 800 µL media were removed and placed on a new tube containing 1 mL 1× cold PBS+100 µg ml^{-1} CHX to stop the reaction. Cells were quickly spun down and washed once more with 1 mL 1× cold PBS+100 µg ml^{-1} CHX. Cells were pelleted, and lyzed in 500 µL polysome buffer for 20 min on ice. Nuclei and mitochondria were cleared by centrifugation at 14,000 r.p.m. at 4 °C for 10 min. Postmitochondrial supernatants (PMS) were placed in new tube and mixed with 500 µL of 0.14 M sucrose in polysome buffer. Half of the sample was used to measure ^{35}S-Met/Cys incorporation into total protein. The rest was centrifuged at 70,000 r.p.m. for 25 min using a TLA 100.3 rotor and the postribosomal supernatant was removed to measure ^{35}S-Met/Cys incorporation in completed polypeptides (PRS). ^{35}S-Met/Cys incorporation was measured using liquid scintillation counting. Counts per units (CPM) were plotted as a function of time, and half transit-times were calculated by linear regression.

Statistical analyses

CLIP-seq:

Mapping: Illumina single ended reads were trimmed of adapter using Cutadapt and any reads less than 21bp were excluded from downstream analysis. The resulting paired-end reads were aligned to four databases using Burrows-Wheeler Aligner (BWA 0.5.5) [2]: (i) human NCBI Build 37 reference sequence, (ii) RefSeq, (iii) a sequence file that represents all possible combinations of non-sequential pairs in RefSeq exons, and (iv) AceView flat file (UCSC), representing transcripts constructed from human expressed sequence tags (ESTs). After this initial mapping, final BAM files were produced by selecting the best alignment in the four databases. Duplicates

were removed. *Coverage:* Effective coverage of the whole genome and whole exome was obtained by summarizing coverage of aligned bases with quality score ≥ 15 at each position of the reference genome (excluding sequencing gaps and ambiguous bases) using the Coverage module of Bambino [3]. Wig files were converted to BigWig files using the UCSC tool; wigToBigWig. *Peak finding:* The genome was split into 100Kb bins with 10Kb overlap and coverage in these windows assessed (using the UCSC tool bigWigSummary) for maximum coverage greater than or equal to the desired cut-off (10). If these criteria were met then this bin was further split into smaller and smaller bins to find the exact peak location. Once this window was small enough base pair by base pair coverage was assessed. Peaks with at least 10 bp long were then refined and merged if required. Peaks present in both the DDX3X and the control IgG CLIPseq were removed. Peaks were manually reviewed using a local mirror of the UCSC genome browser displaying bigWig coverage files as custom tracks to assess their validity.

We used the HOMER (Hypergeometric Optimization of Motif EnRichment)[4] tool "annotatePeaks.pl" to annotate the genomic ontology and gene ontology of each peak. This provided details of the more gross features with peaks and also the possible enrichment of pathways.

RNA-seq:

Mapping: See CLIP-seq mapping. *Expression Analysis:* To compare the expression of genes which contained DDX3X peaks a simple count of the number of reads mapping to that gene was carried out. To compare the expression of genes independently of the CLIPSeq analysis we first obtained counts of the number of reads per gene and carried out FPKM normalization (taking into account gene length and total reads). We applied a log-start transformation to the normalized read counts to minimize the effects of small differences in read counts. Log2ratios were then computed and the average

of each comparison calculated. These results were then plotted in a Magnitude Average plot (MA plot) in a manner similar to microarray analysis.

We also applied a method that utilized exon and exon-exon junction reads. Exon reads were once again normalized by FPKM and exon junctions by the total read count (RPM) and proportional difference values were used in both cases. High confidence results were defined by 10 reads in at least one class.

Ribosome profiling:

Ribosome profiling (Ribo-seq) data analysis was carried out using the same peak detection algorithm as the CLIP-Seq data analysis. Peak statistics based on location in the genome were also determined using the same methods as described in the CLIP-seq section.

References

1 Robinson, G. *et al.* Novel mutations target distinct subgroups of medulloblastoma. *Nature* **488**, 43-48, doi:10.1038/nature11213 (2012).

2 Li, H. & Durbin, R. Fast and accurate short read alignment with Burrows-Wheeler transform. *Bioinformatics* **25**, 1754-1760, doi:10.1093/bioinformatics/btp324 (2009).

3 Edmonson, M. N. *et al.* Bambino: a variant detector and alignment viewer for next-generation sequencing data in the SAM/BAM format. *Bioinformatics* **27**, 865-866, doi:10.1093/bioinformatics/btr032 (2011).

4 Heinz, S. *et al.* Simple combinations of lineage-determining transcription factors prime cis-regulatory elements required for macrophage and B cell identities. *Molecular cell* **38**, 576-589, doi:10.1016/j.molcel.2010.05.004 (2010).

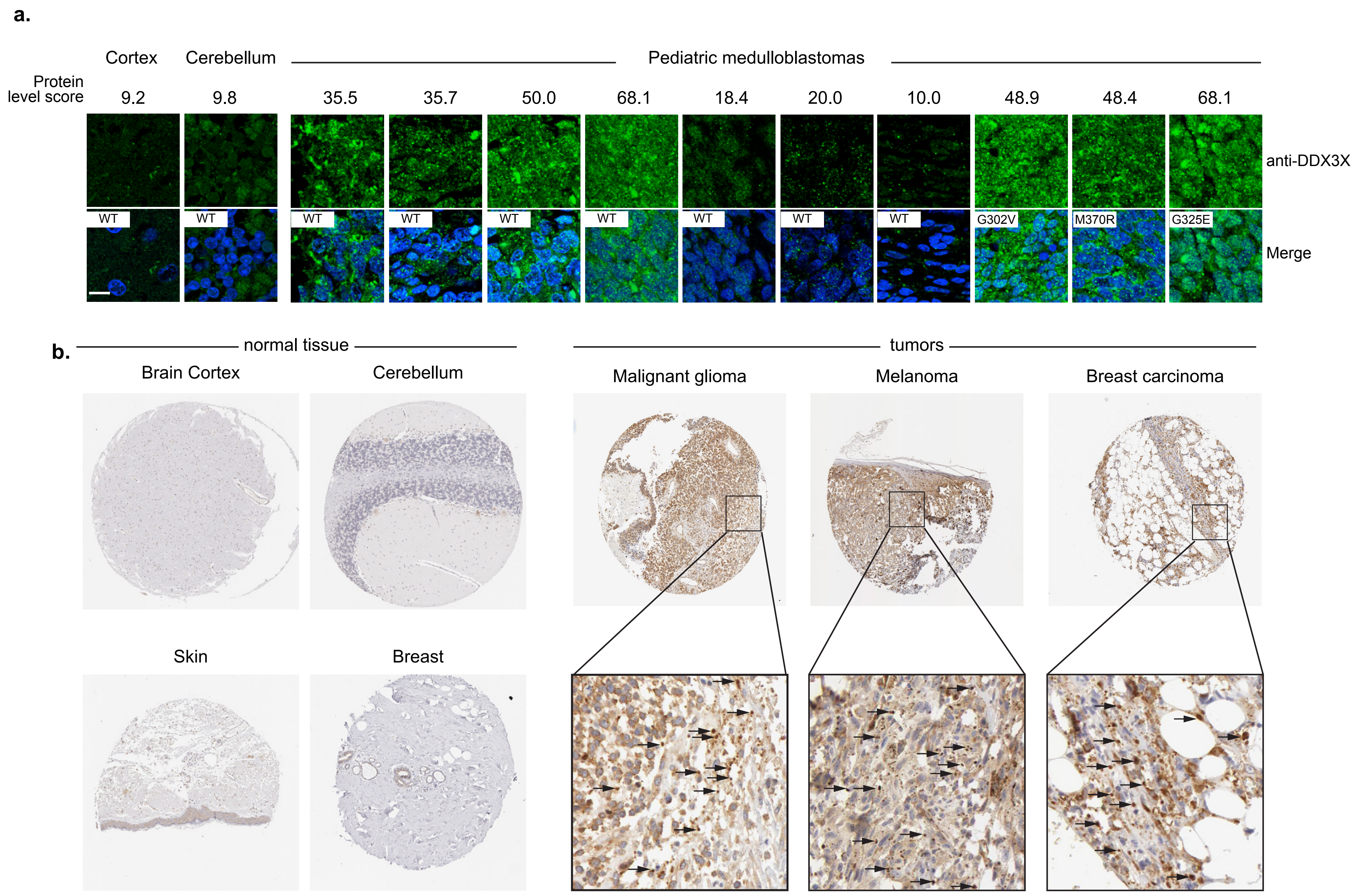

Supplementary figure S1. High expression and punctated distribution of DDX3X in human cancer.

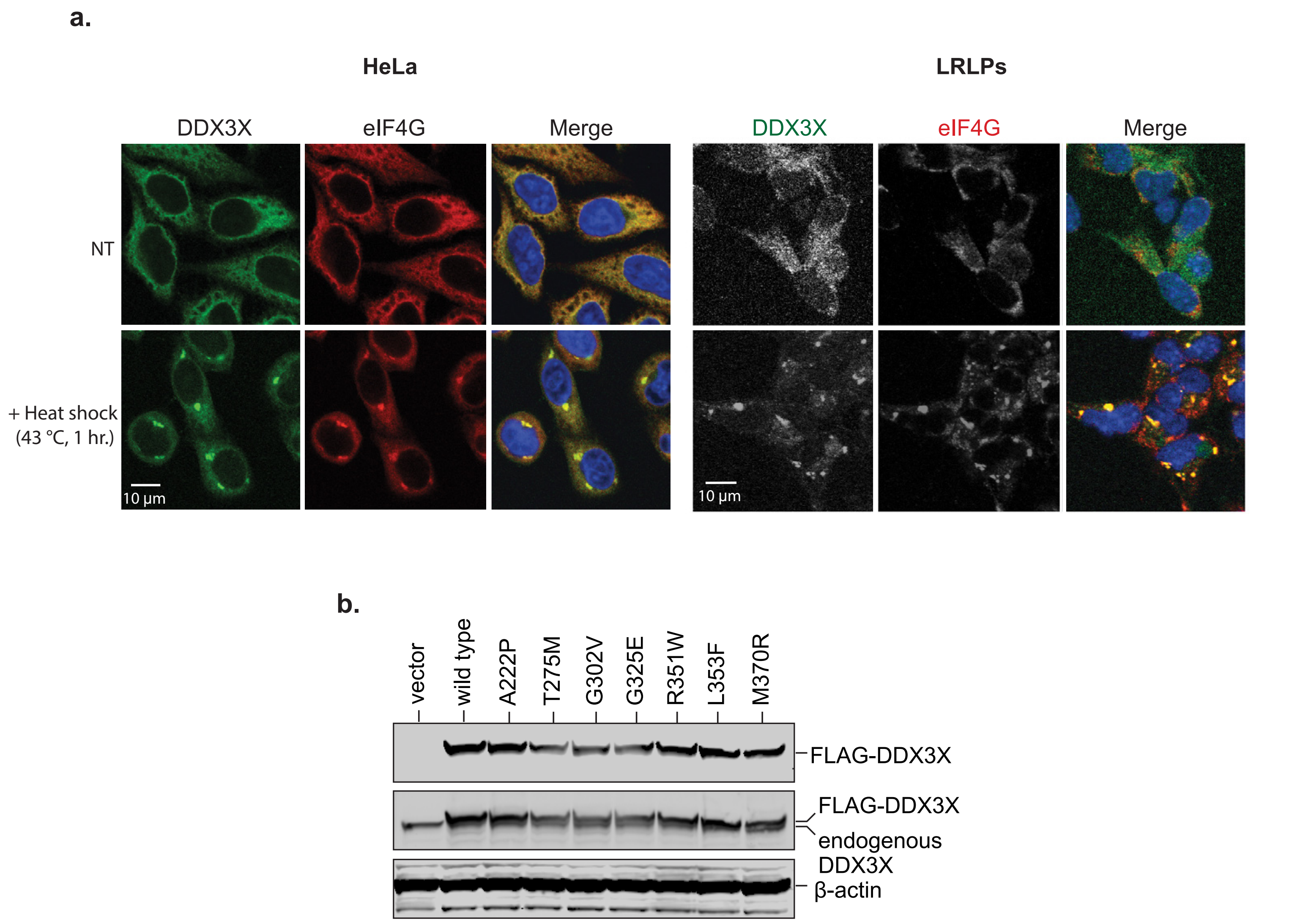

Supplementary figure S2. Medulloblastoma-associated mutations in DDX3X induce stress granule formation.

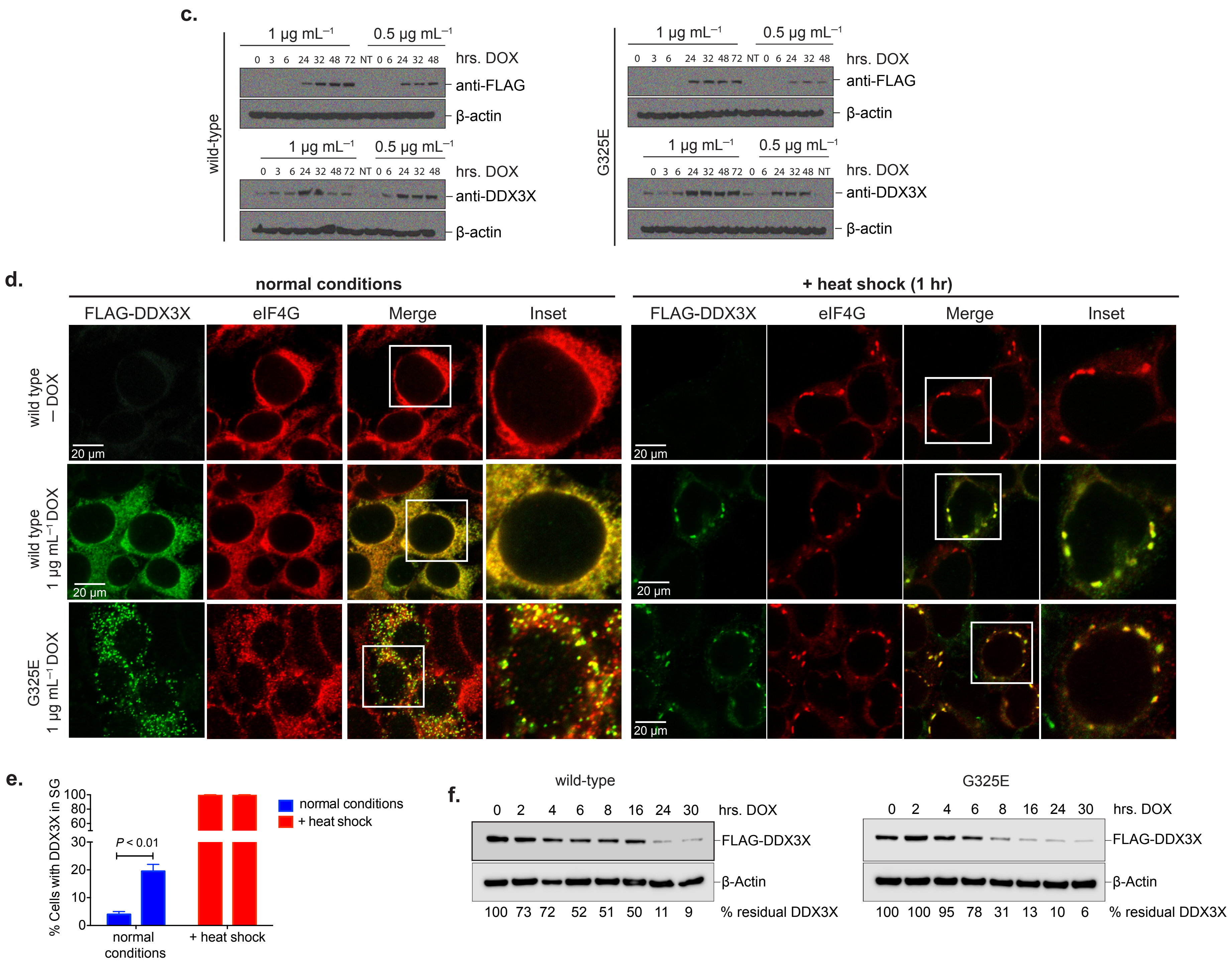

Supplementary figure S2. Medulloblastoma-associated mutations in DDX3X induce stress granule formation.

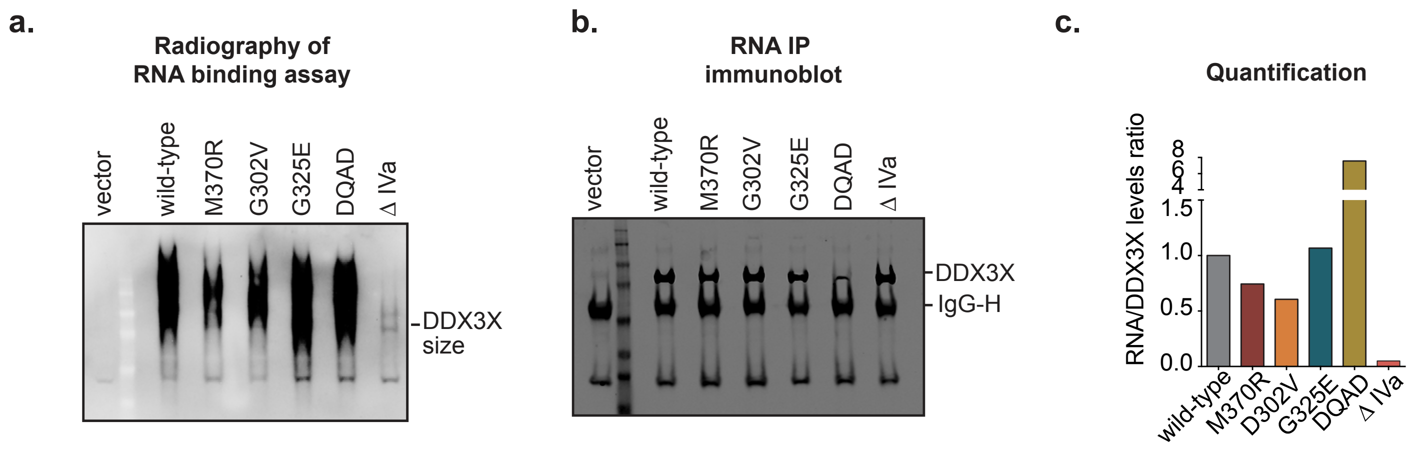

Supplementary figure S3. MB-associated DDX3X mutants are capable of binding RNA *in vivo*.

a.

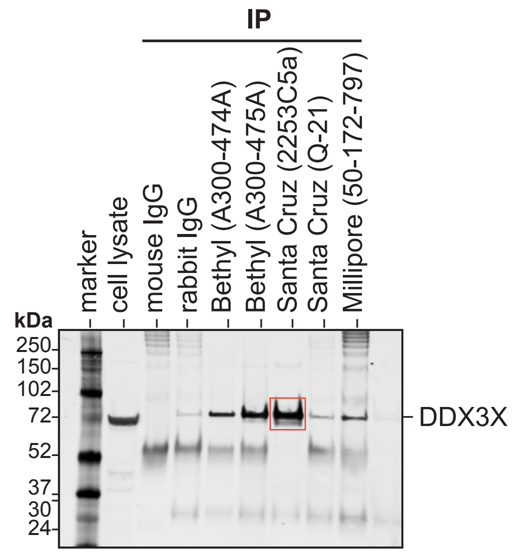

b.

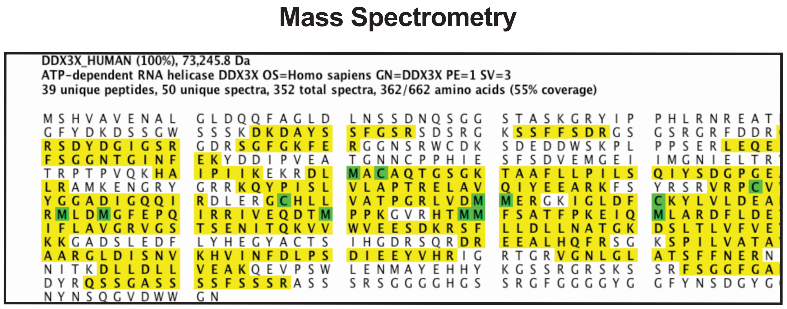

c.

Sample	Reads	Post-trim	Mapped	Un-duplicated mapped	% Mapped
DDX3X CLIP A	66,513,470	9,053,923	2,613,756	209,488	28.90%
DDX3X CLIP B	62,625,544	6,366,983	2,369,216	287,219	37.20%
IgG CLIP	14,191,269	132,948	17,769	1,830	13.40%

d.

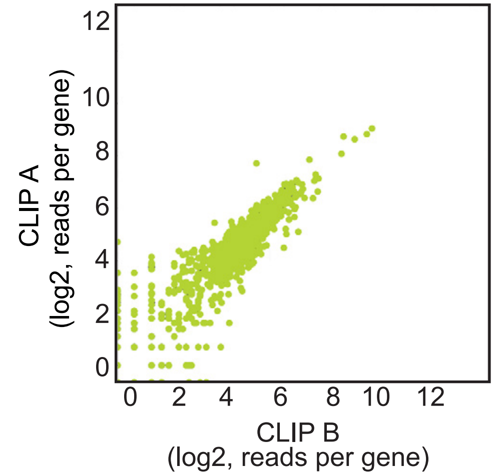

e.

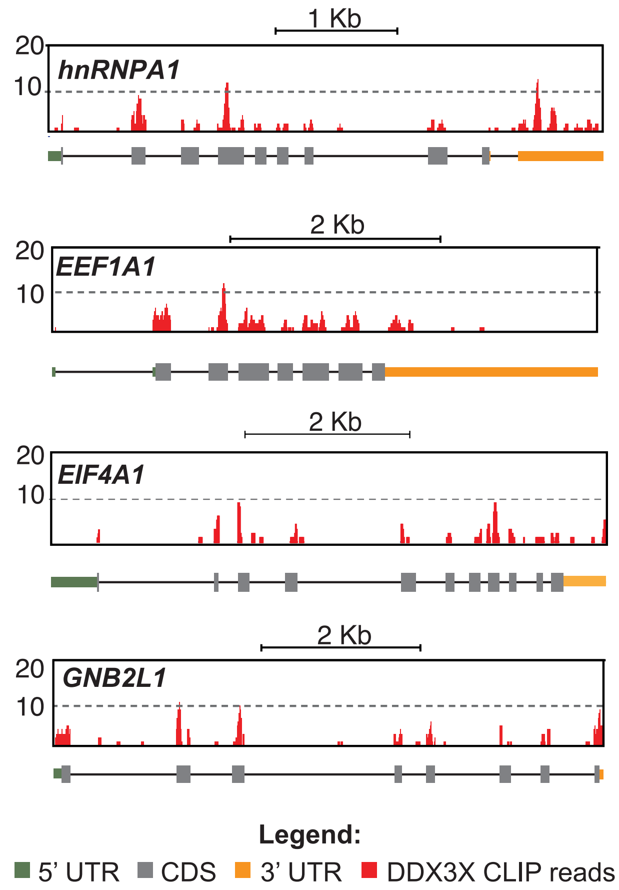

Supplementary figure S4. CLIP-seq of endogenous DDX3X in HEK293T cells.

a.

Sample	Reads	Mapped	Non-dupplicated mapped	% Mapped
ligation control	156,689,321	162,509	20,266	0.1%
vector	139,047,315	32,441,902	797,733	23.3%
wild-type	138,847,639	36,289,083	1,163,463	26.1%
G325E	80,719,271	29,153,123	687,293	33.1%

b.

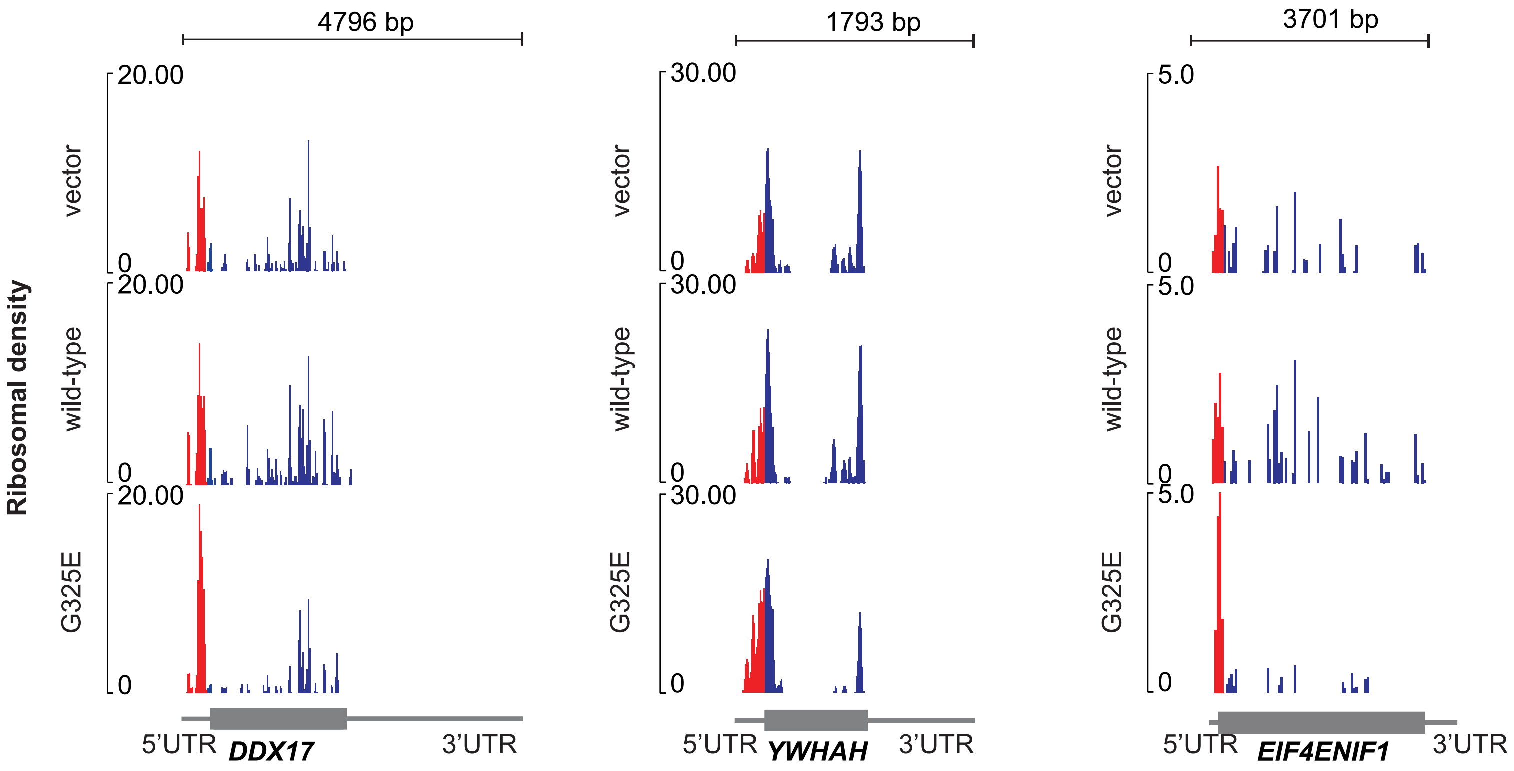

Supplementary figure S5. Ribosomal profiling in HEK293T cells expressing vector, wild-type or MB-associated mutant G325E DDX3X forms.

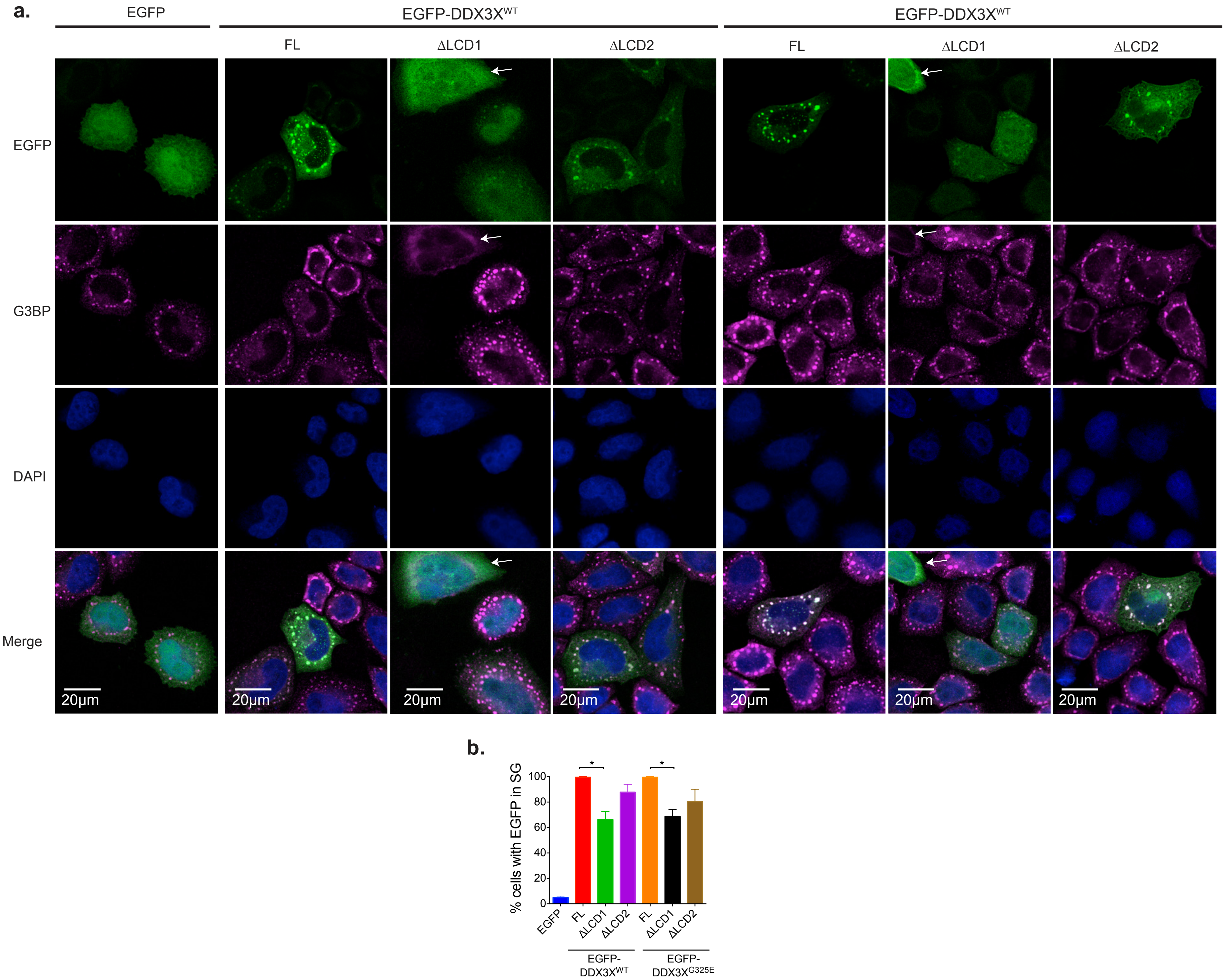
a.
EGFP
EGFP-DDX3X^{WT}
EGFP-DDX3X^{WT}
FL
ΔLCD1
ΔLCD2
FL
ΔLCD1
ΔLCD2
EGFP
G3BP
DAPI
Merge
20μm
b.
% cells with EGFP in SG
100
80
60
40
20
0
*
*
EGFP
FL
ΔLCD1
ΔLCD2
FL
ΔLCD1
ΔLCD2
EGFP-DDX3X^{WT}
EGFP-DDX3X^{G325E}

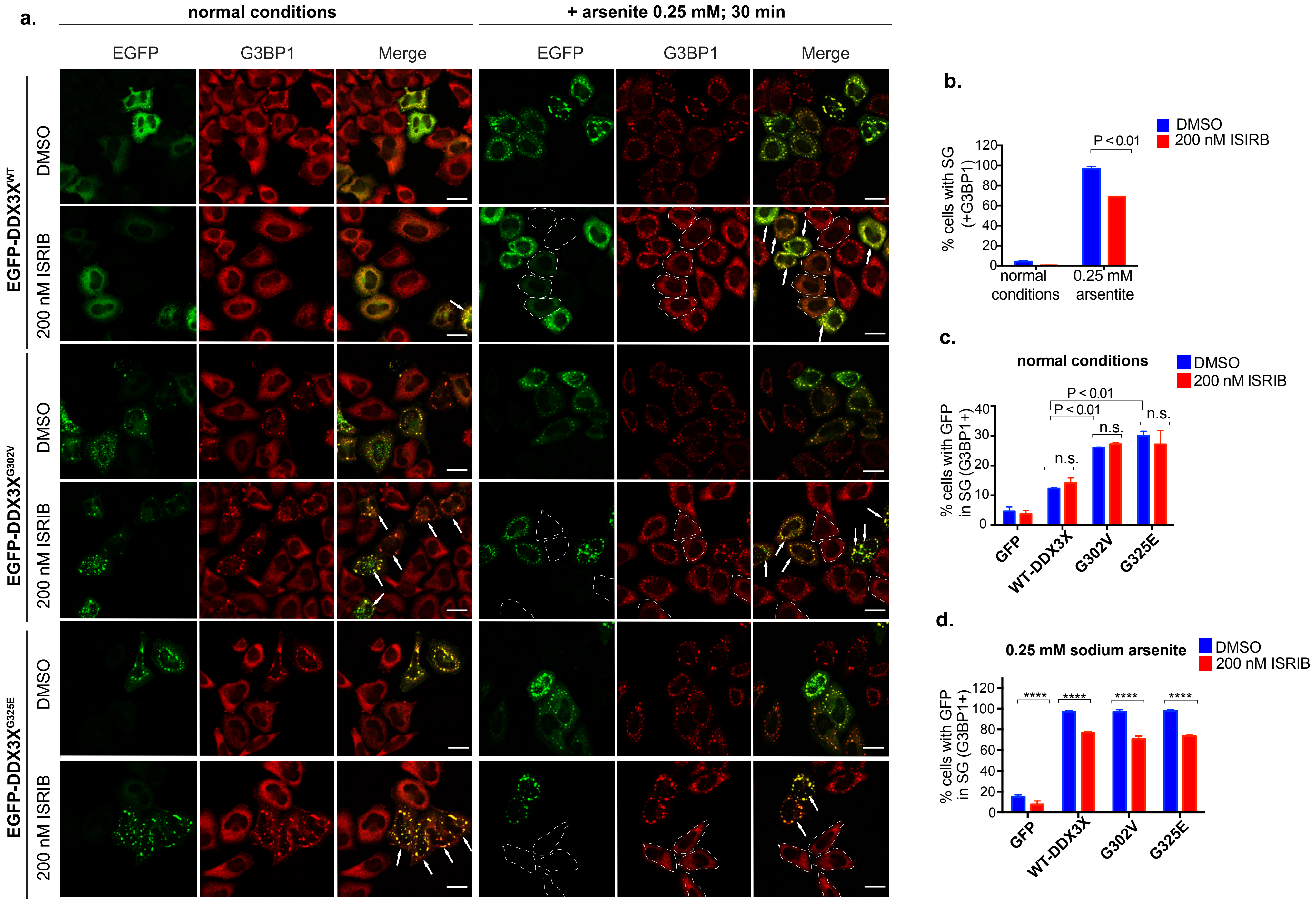

Supplementary Figure S7. Treatment with ISRIB does not prevent cancer-associated mutant DDX3X from inducing SGs.

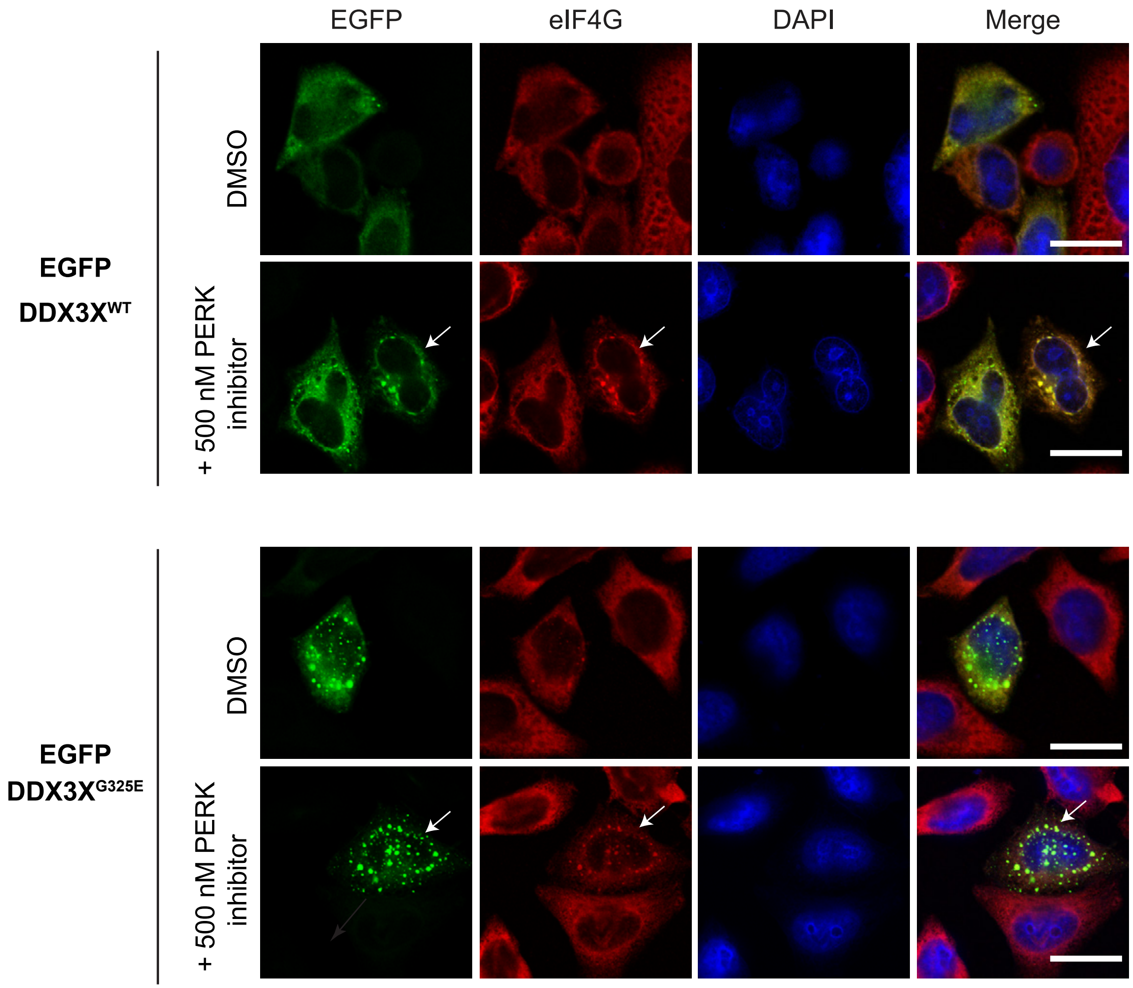

Suplemmentary figure S8: Treatment with the PERK inhibitor GSK2606414 does not prevent cancer-related mutant DDX3X from inducing SG formation.

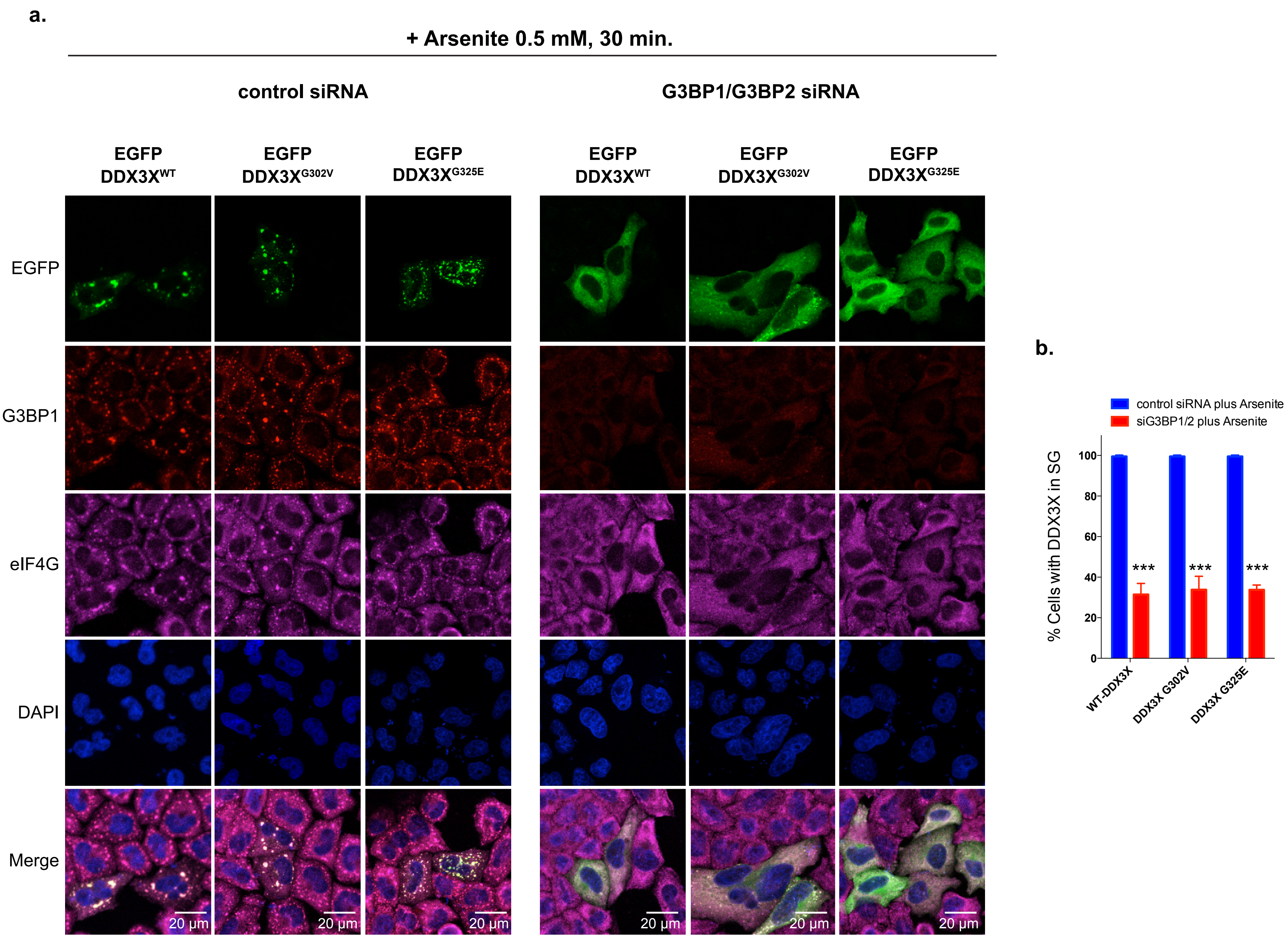

Supplementary figure S9. Knock-down of G3BP1/G3BP2 prevents MB-associated DDX3X mutant from inducing SG formation during stress conditions.
